# Ethylene inhibits rice root elongation in compacted soil via ABA- and auxin-mediated mechanisms

**DOI:** 10.1073/pnas.2201072119

**Published:** 2022-07-18

**Authors:** Guoqiang Huang, Azad Kilic, Michal Karady, Jiao Zhang, Poonam Mehra, Xiaoyun Song, Craig J. Sturrock, Wanwan Zhu, Hua Qin, Sjon Hartman, Hannah M. Schneider, Rahul Bhosale, Ian C. Dodd, Robert E. Sharp, Rongfeng Huang, Sacha J. Mooney, Wanqi Liang, Malcolm J. Bennett, Dabing Zhang, Bipin K. Pandey

**Affiliations:** ^a^Joint International Research Laboratory of Metabolic & Developmental Sciences, State Key Laboratory of Hybrid Rice, SJTU-University of Adelaide Joint Centre for Agriculture and Health, School of Life Sciences and Biotechnology, Shanghai Jiao Tong University, Shanghai, China;; ^b^Future Food Beacon and School of Biosciences, University of Nottingham, LE12 5RD, United Kingdom;; ^c^Laboratory of Growth Regulators, Institute of Experimental Botany of the Czech Academy of Sciences and Faculty of Science of Palacký University, CZ-78371 Olomouc, Czech Republic;; ^d^Biotechnology Research Institute, Chinese Academy of Agricultural Sciences, Beijing 100081, China;; ^e^Plant Environmental Signalling and Development, Faculty of Biology, University of Freiburg, 79104 Freiburg, Germany;; ^f^CIBSS – Centre for Integrative Biological Signalling Studies, University of Freiburg, 79104 Freiburg, Germany;; ^g^Centre for Crop Systems Analysis, Wageningen University & Research, Wageningen, The Netherlands;; ^h^Lancaster Environment Centre, Lancaster University, Lancaster, United Kingdom;; ^i^Division of Plant Science and Technology and Interdisciplinary Plant Group, University of Missouri, Columbia, MO 65211;; ^j^School of Agriculture, Food and Wine, University of Adelaide, Waite Campus, Urrbrae, SA, Australia

**Keywords:** roots, soil compaction, ethylene, auxin, ABA

## Abstract

Soil compaction represents a major agronomic challenge, inhibiting root elongation and impacting crop yields. Roots use ethylene to sense soil compaction as the restricted air space causes this gaseous signal to accumulate around root tips. Ethylene inhibits root elongation and promotes radial expansion in compacted soil, but its mechanistic basis remains unclear. Here, we report that ethylene promotes abscisic acid (ABA) biosynthesis and cortical cell radial expansion. Rice mutants of ABA biosynthetic genes had attenuated cortical cell radial expansion in compacted soil, leading to better penetration. Soil compaction-induced ethylene also up-regulates the auxin biosynthesis gene *OsYUC8*. Mutants lacking OsYUC8 are better able to penetrate compacted soil. The auxin influx transporter OsAUX1 is also required to mobilize auxin from the root tip to the elongation zone during a root compaction response. Moreover, *osaux1* mutants penetrate compacted soil better than the wild-type roots and do not exhibit cortical cell radial expansion. We conclude that ethylene uses auxin and ABA as downstream signals to modify rice root cell elongation and radial expansion, causing root tips to swell and reducing their ability to penetrate compacted soil.

## Significance

Intensive agriculture and changing tillage practices are causing soils to become increasingly compacted. Hard soils cause roots to accumulate the hormone ethylene, triggering reduced root elongation and increased radial swelling. We demonstrate that ethylene regulates these distinct root growth responses using different downstream signals, auxin, and abscisic acid (ABA). Auxin is primarily required to reduce cell elongation during a root compaction response, whereas ABA promotes radial cell expansion. Radial swelling was originally thought to aid root penetration in hard soil, yet rice ABA-deficient mutants disrupted in radial swelling of root tips penetrate compacted soil better than wild-type plants. The combined growth responses to auxin and ABA function to reduce the ability of roots to penetrate compacted soil.

Soil compaction represents a major challenge facing modern agriculture (1), reducing crop yields by ∼25%, and when combined with drought by up to 75%, as roots are challenged to penetrate hard soils (2–4). Europe has over 33 million hectares of soil prone to compaction, which represents the highest in the world as compared to Asia (10 million hectares) and Africa (18 million hectares) (5, 6). Soil compaction inhibits root penetration and decreases uptake of water and nutrients. Efforts to mitigate the impacts of soil compaction on crop productivity include reducing tillage, controlled traffic farming and subsoil management (2, 6). However, these approaches can be time consuming, costly to implement and ineffective for deeper soil profiles.

Breeding crops to better withstand compacted soil offers a genetic solution to improve root growth during mechanical impedance (6). Recently, maize roots featuring small cells with thick walls in outer cortical layers (termed multiseriate cortical sclerenchyma [MCS]) were reported to improve rooting depth in compacted soil in greenhouse and field trials (7). The MCS anatomical trait was associated with greater root lignin concentration, tensile strength and root tip bending force compared to non-MCS genotypes. Conventional thinking considers that roots are unable to penetrate compacted soils because axial root tip growth force exerted by the plant roots is less than mechanical resistance produced by compacted soil (8, 9). However, we recently discovered that plant roots use the gaseous hormone ethylene to sense soil compaction (10). Our experiments revealed that compacted soil restricts diffusion of ethylene out of and away from roots, triggering inhibition of root elongation while also promoting radial swelling (10). Remarkably, roots of *Arabidopsis* and rice mutants that are insensitive to ethylene are able to better penetrate compacted soil (10). Ethylene-response mutant roots remain narrow and able to penetrate compacted soil, whereas wild-type roots exhibit inhibition of cell elongation and promotion of cortical cell radial expansion (10). How ethylene triggers these root growth changes is currently unclear.

Here, we reveal key roles for the hormone signals auxin and abscisic acid (ABA) and their biosynthesis pathways as regulatory targets of the ethylene-response pathway during rice root growth responses to soil compaction. Exogenous treatment with ABA promotes radial expansion of root cortical cells, whereas auxin inhibits root cell elongation. Furthermore, we demonstrate that ABA acts downstream of ethylene and auxin signaling during a soil compaction response. Integrating ethylene, auxin, and ABA responses during a soil compaction response appears to support ABA-mediated radial cell expansion. We conclude that ethylene uses auxin and ABA as downstream signals to modify rice root elongation and radial expansion, disrupting the root’s ability to penetrate compacted soil.

## Results

### Soil Compaction Induces ABA Biosynthetic Gene Expression and Hormone Levels.

**Fig. 1.**
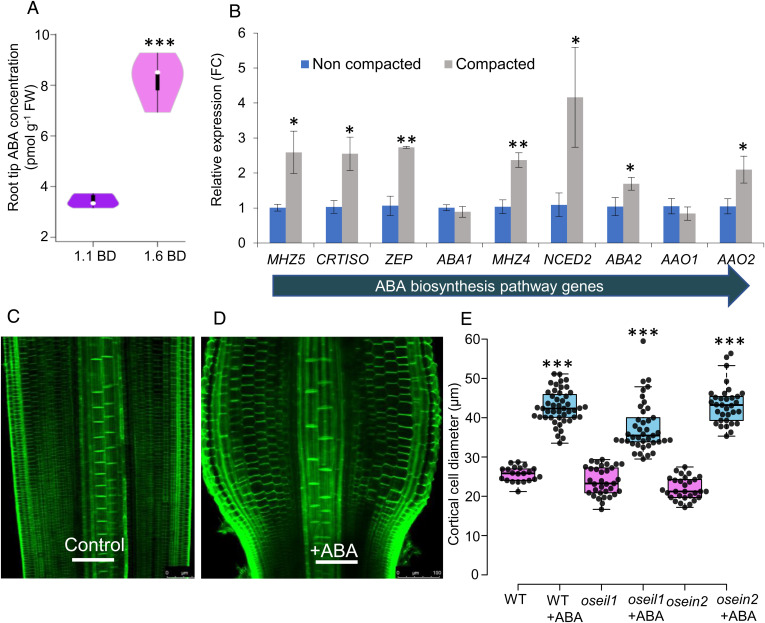
Soil compaction enhances ABA biosynthesis and radial expansion of rice primary root cortical cells. (*A*) Violin plot showing ABA concentration in root tips of wild-type (cv. Nipponbare) grown in noncompacted (1.1 g cm^−3^ BD) and compacted (1.6 g cm^−3^ BD) soil. (*B*) Bar graph showing relative expression (in fold change, FC) of ABA biosynthetic genes in root tips grown in compacted as compared to noncompacted soil, *n* = 3 replicates. Horizontal arrow shows the direction in the pathway of genes encoding within the ABA biosynthetic pathway in rice. (*C*, *D*) Representative images of control and ABA-treated root tips (elongation zone) showing radial expansion of root cortical cells after ABA treatment (10 µM ABA for 24 h); green fluorescence represents staining with calcofluor dye. Scale bars represent 100 µm. (*E*) Quantitative box plot showing the radial expansion (diameter of cortical cells) of ABA-treated root tips of wild-type (WT), *oseil1* and *osein2* lines. *, **, and *** represent *P* values of ≤ 0.05, 0.01, and 0.001, respectively, calculated by Student’s *t* test between compacted and noncompacted treatments (*A*, *B*) and between genotypes with or without ABA treatment (*E*).

Several hormone signals have been reported to function downstream of ethylene (11–14) to inhibit root elongation, including auxin and ABA (15–17). However, the interaction of these hormones has not been studied in roots during growth in compacted soil. To address this point, we initially measured ABA levels in rice primary root tip tissues from plants grown in either noncompacted (1.1 g cm^−3^ bulk density [BD]) or compacted (1.6 g cm^−3^ BD) soil conditions using ultra-high performance liquid chromatography-electrospray tandem mass spectrometry (UHPLC-MS/MS). ABA levels increased threefold in root tips growing in compacted versus noncompacted soil conditions (Fig. 1*A*).

To reveal why root tips grown in compacted soil have increased ABA levels, we examined the effect of soil compaction on transcript abundances of nine ABA biosynthetic genes (*CRTISO, MHZ5, ABA1, ABA2, MHZ4, AAO1, AAO2, NCED2* and* ZEP*) in root tip tissues (Fig. 1*B*). Transcript analysis revealed that the majority (seven out of nine) of the ABA biosynthetic genes profiled were up-regulated in wild-type root tips grown in compacted *versus* noncompacted soil conditions. In contrast, none of the ABA biosynthetic genes were up-regulated significantly in root tips of the ABA biosynthetic mutant *mhz5-1* grown in compacted soil conditions (*SI Appendix*, Fig. S1*A*). Expression of ABA biosynthesis genes has been reported to be subject to positive feedback regulation by ABA (18), suggesting that mutating *MHZ5* may attenuate positive up-regulation of other biosynthesis genes by ABA.

### ABA Biosynthesis Is Up-Regulated by Ethylene during a Root Compaction Response.

To examine the regulatory relationship between ethylene and ABA during root responses to compaction, we treated the wild-type (cv. Nipponbare) and ethylene-insensitive mutants *oseil1* and *osein2* (19) with either ethylene or ABA. While roots of the *oseil1* and *osein2* mutants did not respond to ethylene treatment, width of wild-type roots doubled largely due to radial expansion of cortical cells (*SI Appendix*, Fig. S2 *A*–*G*). In contrast, ABA treatment caused cortical cells of all three lines to undergo radial expansion, mimicking the effects of ethylene on the wild-type roots (see Fig. 1*D* versus control in Fig. 1*C* for wild-type images, plus mutant images in *SI Appendix*, Fig. S3; cortical cell diameters ± ABA treatments are shown in Fig. 1*E*). This observation suggested that ABA can trigger cortical cell radial expansion, and this signal acts downstream of the ethylene pathway in modulating root growth in response to soil compaction. To validate that ABA acts downstream of ethylene during a root compaction response, we examined the *osein2 mhz5-1* double mutant and *OsEIN2 OE mhz5-1* (*OsEIN2* overexpressing line crossed with ABA biosynthetic mutant *mhz5-1*) in compacted and noncompacted soil conditions. Our results revealed that neither line exhibited cortical cell radial expansion in compacted soil (*SI Appendix*, Fig. S4 *A*–*F*), validating that ABA acts downstream of ethylene during a compaction response and is required for radial expansion of cortical cells.

To determine how ethylene regulates ABA levels, we examined the expression of ABA biosynthetic genes in wild-type rice root tips treated with ethylene. Ethylene was observed to elevate transcript levels of the ABA biosynthesis genes profiled (*MHZ5, CRTISO* and* ZEP*; *SI Appendix*, Fig. S5), consistent with when roots are exposed to soil compaction conditions and the enhanced ethylene response promoted ABA biosynthesis (Fig. 1*B*). To understand in which tissues ABA biosynthetic genes were up-regulated by ethylene during a compaction response, we performed *in-situ* hybridization experiments on *MHZ4* and *MHZ5*. This revealed induction of ABA biosynthesis genes in response to compacted soil conditions in root vascular tissues (*SI Appendix*, Fig. S6 *A*–*H*).

### ABA Promotes Radial Expansion of Root Cortical Cells under Compacted Conditions.

**Fig. 2.**
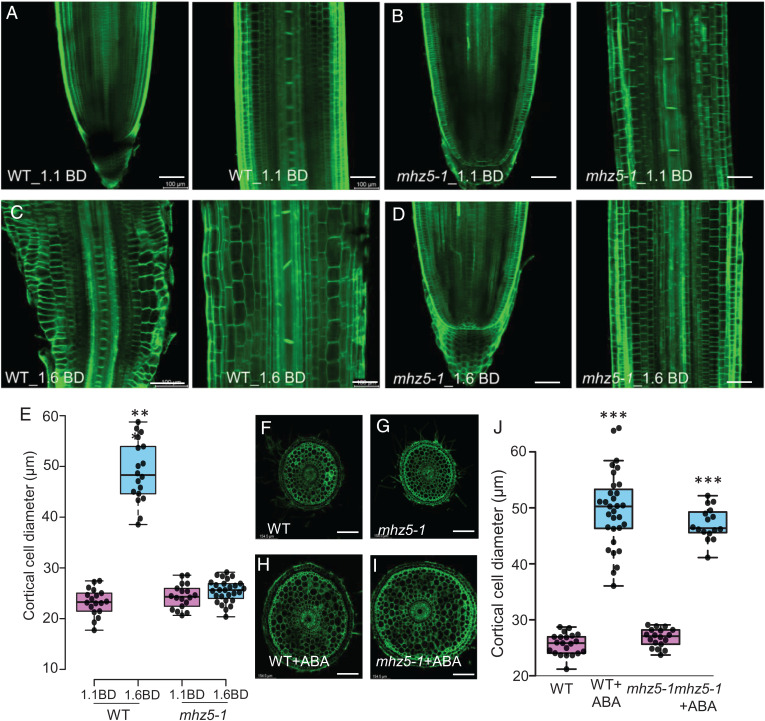
ABA promotes radial expansion of rice primary root cortical cells in compacted soil. (*A*–*D*) Representative images of wild-type (WT) and *mhz5-1* (ABA-deficient mutant) root tips (meristematic and elongation zones) showing cortical cells in (*A*, *B*) noncompacted soil (1.1 g cm^−3^ BD) as compared with (*C*, *D*) compacted soil (1.6 g cm^−3^ BD). Scale bars = 100 µm. (*E*) Boxplot showing the radial expansion of root cortical cells in wild-type and *mhz5-1* root tips (elongation zone) in noncompacted and compacted soils. (*F*–*I*) Representative radial sections of wild-type and *mhz5-1* root tips grown under control (*F*, *G*) and 10 µM ABA (*H*, *I*) treatments for 48 h. Scale bar = 154.5 µm. (*J*) Boxplot of wild-type and *mhz5-1* root tips showing the radial expansion of root cortical cells after ABA treatment. All experiments were repeated three times. *** represents *P* value of ≤ 0.0001 calculated by Student’s *t*-test between noncompacted and compacted treatments (*E*) and between control and ABA treatments (*J*).

To determine whether ABA is essential for radial expansion of root cortical cells in compacted soil conditions, we examined the phenotypes of several rice ABA biosynthetic mutants. *MHZ5* encodes an ethylene-inducible ABA biosynthetic enzyme (*SI Appendix*, Fig. S5) which, when mutated, displays an ethylene-insensitive root growth phenotype (20). Wild-type and *mhz5-1* lines were grown in either noncompacted (1.1 g cm^−3^ BD) or compacted (1.6 g cm^−3^ BD) soil conditions, then excavated roots were radially sectioned and confocal imaged (see *Materials and Methods*). In contrast to the wild-type (Fig. 2*A* and *C*), root diameter of *mhz5-1* did not significantly increase when grown in compacted soil (Fig. 2*B* and *D*). Cortical cell width of wild-type roots doubled under compacted soil conditions (Fig. 2*C* and *E*), which was blocked in *mhz5-1* roots (Fig. 2*D* and *E*). Nevertheless, external treatment with ABA in a hydroponic system induced radial expansion of cortical cells in *mhz5-1* (and wild-type) roots by more than two-fold (Fig. 2*F*–*J*), confirming that *mhz5-1* remains sensitive to ABA. We also confirmed that ABA promotes root radial expansion in soil conditions. Treatments using a series of ABA concentrations (0, 10, 50, and 100 µM ABA) to wild-type roots grown in noncompacted soil (*SI Appendix*, Fig. S7*A*) revealed that 10 µM ABA is sufficient to mimic soil compaction responses (*SI Appendix*, Fig. S7 *C*–*F*). Furthermore, *mhz5-1* remains sensitive to ABA in noncompacted soil conditions and exhibits a similar response (more than twofold increase in cortical cell diameter) as the wild-type roots (*SI Appendix*, Fig. S8 *A*–*E*). We conclude that ABA biosynthesis is required to promote root radial expansion in response to soil compaction conditions (Fig. 2*J*).

### ABA Biosynthetic Mutants Exhibit Increased Root Penetration in Compacted Soil.

**Fig. 3.**
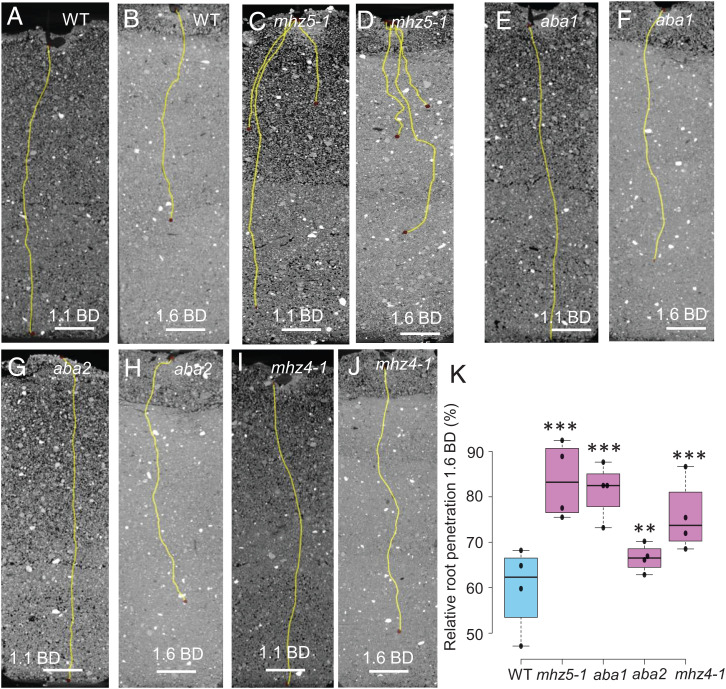
ABA-deficient rice mutants have better root penetration ability than wild-type in compacted soil. Representative CT images of primary root length in (*A*, *B*) wild-type (WT; cv. Nipponbare) as compared with (*C*, *D*) *mhz5-1*, (*E*, *F*) *osaba1*, (*G*, *H*) *osaba2*, and (*I*, *J*) *mhz4-1* ABA-deficient mutants in noncompacted (1.1 g cm^−3^ BD) and compacted (1.6 g cm^−3^ BD) soil. (*K*) Box plot showing root penetration ability of wild-type and *mhz5-1, osaba1, osaba2*, and *mhz4-1* mutants in compacted soil expressed as % of root growth in noncompacted soil. For CT imaging, four replicates were imaged, and the experiment was repeated twice. ** and *** represent *P* values of ≤ 0.01 and 0.001, respectively, calculated by Student’s *t*-test. Scale bars in *A*–*J* represent 10 mm.

Next, we studied the impact of disrupting ABA biosynthesis on root penetration in compacted soil. To examine this, we performed noninvasive X-ray micro-computed tomography (CT) imaging (21) of wild-type and *mhz5-1* roots grown in noncompacted and compacted soil conditions (Fig. 3*A*–*D*). CT imaging in compacted soil revealed that *mhz5-1* root elongation was much less inhibited (∼15% reduction) compared to wild-type (∼40%) (Fig. 3*K*). Hence, cortical cell swelling in response to soil compaction does not aid root penetration capacity. Instead, ABA-dependent root swelling appears to reduce root elongation when exposed to compacted soil.

To independently validate this, we examined the root penetration ability of several other rice ABA biosynthetic mutants (Fig. 3 and *SI Appendix*, Fig. S1*B*) (22–24). The *osaba1* mutant is unable to catalyze oxidation of zeaxanthin to produce transviolaxanthin and has much less ABA than the wild-type plants (22), while the *osaba2* mutant is unable to produce abscisic aldehyde that is converted to ABA by AAO (abscisic aldehyde oxidase) (23). The *mhz4* mutant, which also contains less ABA (61% of wild-type), is homologous to the *Arabidopsis aba4* mutant (24). *Arabidopsis ABA4* encodes neoxanthin synthase, which converts transviolaxanthin to neo-violaxanthin, and the *aba4* mutant does not accumulate ABA under water-deficit stress (25). CT imaging revealed that all of the ABA biosynthetic mutants examined (*osaba1*, *osaba2*, *mhz4*, and *mhz5-1*) exhibited higher root penetration ability than wild-type roots in compacted soil (Fig. 3*E*–*K*). Hence, our mutant analysis confirmed that ABA functions to reduce root penetration in compacted soil conditions.

To probe the role of ABA in regulating root penetration activity, we quantified ABA levels in root tips of the ABA biosynthetic mutants grown in noncompacted (1.1 g cm^−3^ BD) and compacted soil (1.6 g cm^−3^ BD) conditions. UHPLC-MS/MS analysis revealed that, in contrast to wild-type, compacted soil conditions did not induce significantly higher ABA levels in the mutants (*SI Appendix*, Fig. S1*C*). ABA biosynthetic mutants also exhibited less radial expansion of cortical cells than the wild-type roots in compacted soil conditions (*SI Appendix*, Fig. S9 *A*–*H*). By comparing ABA accumulation (*SI Appendix*, Fig. S1*C*) and radial expansion (*SI Appendix*, Fig. S9 *A*–*I*) datasets, we conclude that higher ABA levels correlate with greater radial expansion of cortical cells (and reduction in root penetration ability) in compacted soil conditions.

### Ethylene Inhibits Root Elongation during Soil Compaction via Auxin.

**Fig. 4.**
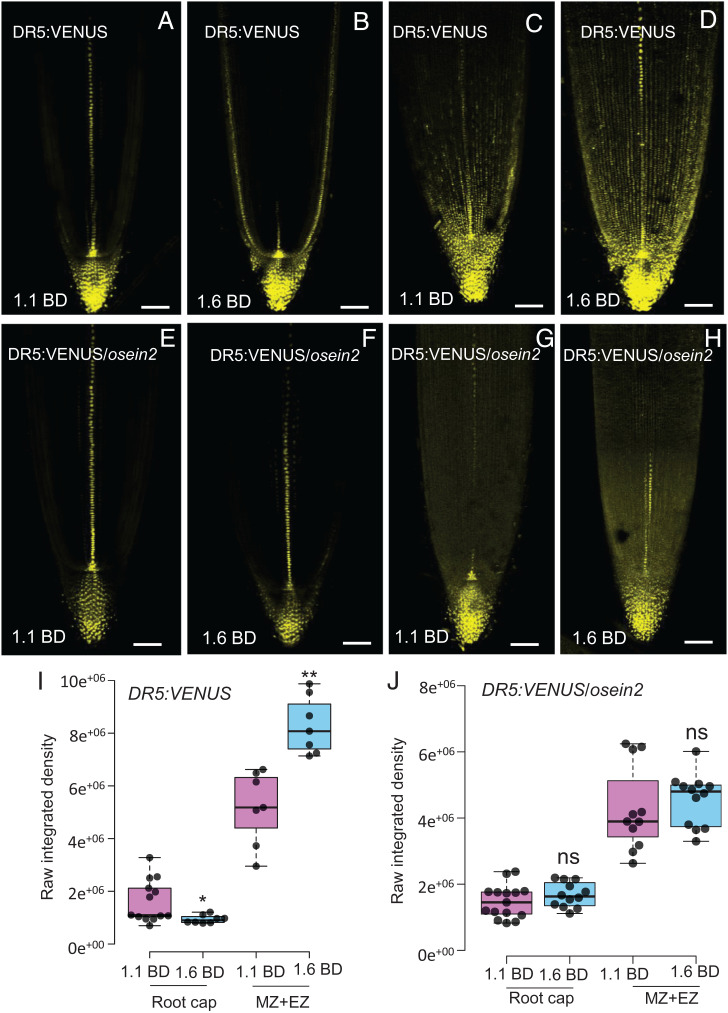
Soil compaction triggers higher auxin response in rice primary root epidermal cells to restrict epidermal cell expansion. (*A*–*D*) Representative confocal images [at median plane (*A*, *B*) and maximum projection (*C*, *D*)] of primary root tips of rice auxin reporter (*DR5:VENUS*) seedlings grown in (*A*, *C*) noncompacted (1.1 g cm^−3^ BD) and (*B*, *D*) compacted (1.6 g cm^−3^ BD) soil. (*E*–*H*) Representative confocal images [at median plane (*E*, *F*) and maximum projection (*G*, *H*)] of primary root tips of rice auxin reporter (*DR5:VENUS*) crossed with *osein2* mutant seedlings grown in (*E*, *G*) noncompacted and (*F*, *H*) compacted soil. Scale bars in *A*–*H* represent 100 µm. (*I*) Box plot showing the quantitative VENUS signal in the root cap and meristematic (MZ) plus elongation (EZ) zones of *DR5:VENUS* primary roots grown in 1.1 g cm^−3^ and 1.6 g cm^−3^ BD soil. (*J*) Box plot showing the quantitative VENUS signal in the root cap and meristematic (MZ) plus elongation (EZ) zones of the primary root in the *ein2* mutant background grown in 1.1 g cm^−3^ BD and 1.6 g cm^−3^ BD soils. * and ** represent *P* values of ≤ 0.05 and 0.001, respectively, calculated by Student’s *t*-test.

**Fig. 5.**
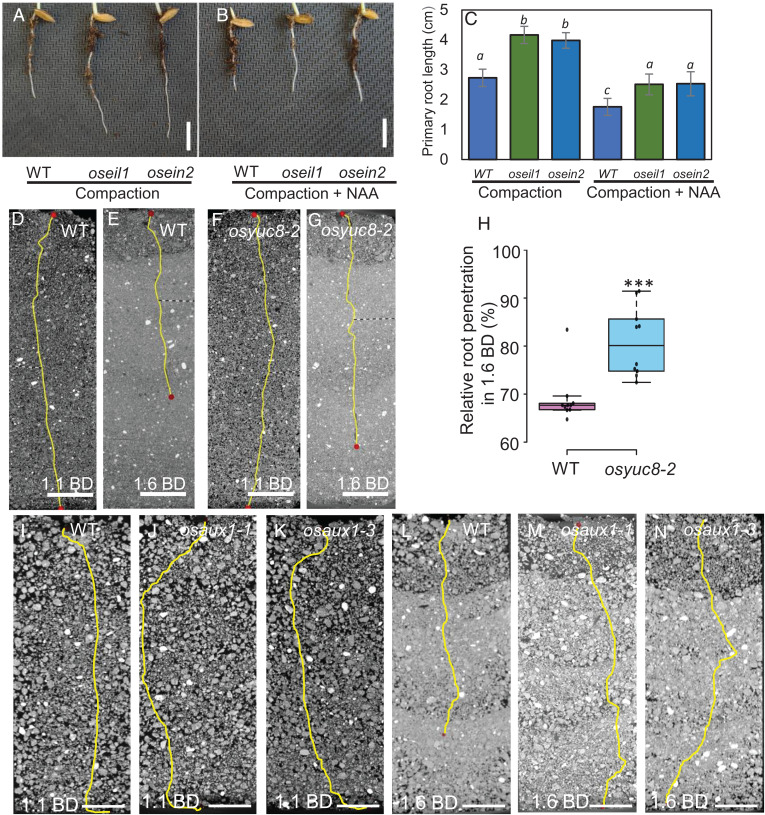
Mutations in *OsYUC8* and *osaux1* confer higher penetration of rice primary roots in compacted soil. (*A*–*C*) Phenotypes of wild-type (WT; cv. Nipponbare) and *oseil1* and *osein2* mutant roots grown in compacted soil (1.6 g cm^−3^ BD) without (*A*) and with (*B*) addition of 100 nM NAA (synthetic auxin analog) and their corresponding primary root lengths (*C*). Scale bars represent 1 cm. Data are means ± SD, *n* = 10. Different letters indicate significant differences (*P* < 0.01, Student’s *t* test), comparing *oseil1* and *osein2* root lengths in compacted conditions without and with NAA treatment to the respective wild-type values. (*D*–*G*) Representative CT images of (*D*, *E*) wild-type (WT; cv. Hwayoung and (*F*, *G*) *yuc8-2* (auxin biosynthesis mutant) primary root length in noncompacted (1.1 g cm^−3^ BD) and compacted (1.6 g cm^−3^ BD) soil. (*H*) Box plot showing root penetration ability of wild-type and *Osyuc8-2*, mutant in compacted soil (1.6 g cm^−3^ BD) expressed as % of root growth in noncompacted (1.1 g cm^−3^ BD) soil. This experiment was repeated three times and the final experiment was performed using CT imaging. *** represent *P* values of ≤ 0.0005, calculated by Student’s *t*- test. Scale bars in *D*–*G* represent 15 mm. (*I*–*N*) Representative CT images of wild-type (cv. Dongjin) and *osaux1-1*, *osaux1-3* mutants (auxin influx carrier mutant alleles) grown in noncompacted (*I*–*K*) (1.1 g cm^−3^ BD) and (*L*–*N*) compacted (1.6 g cm^−3^ BD) soil. Scale bars represent 10 mm.

Ethylene has been reported to inhibit root growth by elevating auxin levels in *Arabidopsis thaliana* (11, 13, 26), but the role of auxin during soil compaction conditions is unclear. To test whether and where auxin response is altered in rice roots grown in compacted soils, we imaged the roots expressing the *DR5:VENUS* auxin reporter (27, 28). Confocal imaging revealed that auxin response was significantly increased in root epidermal cells of wild-type (cv. Nipponbare) roots, specifically in the meristematic and elongation zones, when grown in compacted soil (1.6 g cm^−3^ BD) (Fig. 4, compare *A* to *B* for median plan view and *C* to *D* for maximum projection images). Although there was a slight reduction of auxin response in the root cap in compacted as compared to noncompacted conditions (Fig. 4*I*), there was a significant increase of auxin response specifically in expanding epidermal cells of roots grown in compacted soil (Fig. 4*I*). In contrast, when the *DR5:VENUS* reporter was expressed in the ethylene-insensitive mutant *osein2*, auxin response was unchanged when grown in compacted compared to noncompacted soil conditions (Fig. 4, compare *E* to *F* for median plan view and *G* to *H* for maximum projection images, and Fig. 4*J* and *SI Appendix*, Fig. S10). Consistently, root elongation of the *osein2* and *oseil1* mutants was much less inhibited by soil compaction compared to the wild-type roots [Fig. 5*A* and *C* and ref. (10)]. Our observations suggest that auxin acts downstream of ethylene to mediate inhibition of root elongation during soil compaction.

To directly test the role for auxin under compacted soil conditions, we examined root elongation of wild-type (cv. Nipponbare) and ethylene-response mutants *oseil1* and *osein2* in compacted soil with and without cotreatment with 1-naphthaleneacetic acid (NAA, a synthetic auxin analog). Consistent with auxin acting downstream of ethylene signaling, exogenous NAA application restored root elongation inhibition in *oseil1* and *osein2* mutants under compacted soil conditions (Fig. 5*B* and *C*). However, external NAA application to *oseil1* and *osein2* mutant roots in compacted soil did not induce radial swelling of cortical cells (<20%) as compared to ABA treatment (more than twofold) (*SI Appendix*, Fig. S11). Hence, ethylene inhibits root elongation via auxin during soil compaction stress.

### Ethylene Up-Regulates Auxin Synthesis via OsYUC8 during Compaction Conditions.

To determine how ethylene may up-regulate auxin during soil compaction stress, we functionally examined auxin-related targets of the primary ethylene response pathway. The ethylene-response transcription factor OsEIL1 has been shown to directly bind the promoter of the auxin biosynthesis gene *OsYUC8* (*SI Appendix*, Fig. S12*G*) to regulate ethylene-mediated root elongation (29). Consistent with this previous report, root elongation of the loss-of-function mutant allele *osyuc8-2* was unresponsive to the ethylene precursor ACC (1-aminocyclopropane-l-carboxylic acid) compared with its wild-type background (var. Hwayoung [HY]) (*SI Appendix*, Fig. S12 *A*–*E*). To reveal whether ethylene drives *OsYUC8* up-regulation during root responses to soil compaction, *OsYUC8* transcript abundance was quantified in wild-type, *oseil1*, and *osein2* roots grown in compacted and noncompacted soils. *OsYUC8* transcript levels were enhanced in wild-type roots grown in compacted soil (*SI Appendix*, Fig. S12*F*), but no difference is observed in *osein2* and *oseil1* roots between compacted and noncompacted conditions (*SI Appendix*, Fig. S12*F*). Hence, *OsYUC8* expression was up-regulated in roots grown in compacted soil in an ethylene-dependent manner.

To functionally validate the role of *OsYUC8* during the root compaction response, we phenotyped the loss-of-function mutant *osyuc8-2* versus its wild-type background (cv. Hwayoung). CT imaging revealed that *osyuc8-2* roots grown in compacted soil exhibited less inhibition of elongation than wild-type roots (Fig. 5*D*–*H*), which is similar to *oseil1* lines (Fig. 5*C*), indicating that OsEIL1 is epistatic to *OsYUC8*. Hence, the ethylene (and OsEIL1)-regulated root response to compaction appears to rely on OsYUC8-mediated auxin biosynthesis.

### Root Compaction Response Requires OsAUX1-Mediated Auxin Mobilization.

*OsEIL1* and *OsYUC8* are mainly expressed in inner tissues of the root tip (10, 29), yet auxin response is elevated in the epidermis of roots grown in compacted soil (Fig. 4*A*–*D*). How does OsYUC8-derived auxin reach the epidermal cell layer? The *Arabidopsis* auxin influx carrier AtAUX1 plays a key role in ethylene-mediated inhibition of root elongation by facilitating auxin transport from the apex to the elongation zone (11, 13, 26). To assess the role of the orthologous *OsAUX1* during rice root compaction responses, we characterized the *osaux1-1* and *osaux1-3* T-DNA insertion lines (30, 31). Roots of *osaux1-3* were less sensitive to ACC than the wild-type (cv. Dongjin) (*SI Appendix*, Fig. S13 *A* and *B*), suggesting that *OsAUX1* plays a key role in mediating ethylene-dependent inhibition of rice root elongation. CT imaging revealed that in contrast to the inhibition of root elongation in the wild-type, root lengths of both *osaux1* mutant alleles were unchanged under compacted soil conditions (Fig. 5*I*–*N* and *SI Appendix*, Fig. S13*C*). Hence, the auxin influx carrier OsAUX1 appears essential for mobilizing auxin to its target root tissue(s) to mediate the inhibition of elongation in response to soil compaction conditions.

To reveal which root tissue(s) are targeted by auxin via OsAUX1 during the root compaction response, we analyzed compaction-induced changes in the spatial expression of the *DR5:VENUS* auxin reporter in wild-type and *osaux1-3* backgrounds. Increased fluorescence intensity of the *DR5:VENUS* reporter in epidermal cells grown in compacted soil was not observed in *osaux1-3* roots (*SI Appendix*, Fig. S14 *A*–*E*). Hence, much less shootward (i.e., root apex to base) auxin transport appears to occur in *osaux1-3* versus wild-type roots, resulting in a reduced auxin response in epidermal cells of the meristematic and elongation zones (*SI Appendix*, Fig. S14 *A*–*E*). To understand the functional consequences of altered auxin response in this outermost root tissue, we measured epidermal cell lengths of wild-type and *osaux1* roots grown in compacted and noncompacted soils. Epidermal cell lengths were significantly reduced in wild-type roots when grown in compacted soil, whereas the *osaux1-1* and o*saux1-3* lines exhibited much less difference between the compaction treatments (*SI Appendix*, Fig. S13 *D* and *E*). Hence, OsAUX1-dependent root compaction responses are associated with increasing shootward auxin transport to epidermal cells of the elongation zone, inhibiting cell and thus root elongation.

Does auxin also play a role in root radial expansion? No change was observed in *osaux1* cortical cells in compacted soil (*SI Appendix*, Fig. S15 *A*–*C*), consistent with the ethylene-mediated disruption in induction of key ABA biosynthesis genes (*SI Appendix*, Fig. S16). However, ABA treatment was able to promote radial expansion of cortical cells in *osaux1* and *osyuc8-2* mutant roots (*SI Appendix*, Figs. S17 and S18). Nevertheless, we noted that ABA did not fully restore *osaux1* radial expansion to a wild-type level, suggesting that auxin (delivered via OsAUX1) may function to modify root epidermal properties to enable cortical expansion in response to ABA (*SI Appendix*, Fig. S17). In summary, we conclude that a compaction-induced ethylene response uses auxin and ABA as downstream signals to modify rice root elongation and radial expansion, causing root tips to swell and reducing their ability to penetrate compacted soil.

## Discussion

**Fig. 6.**
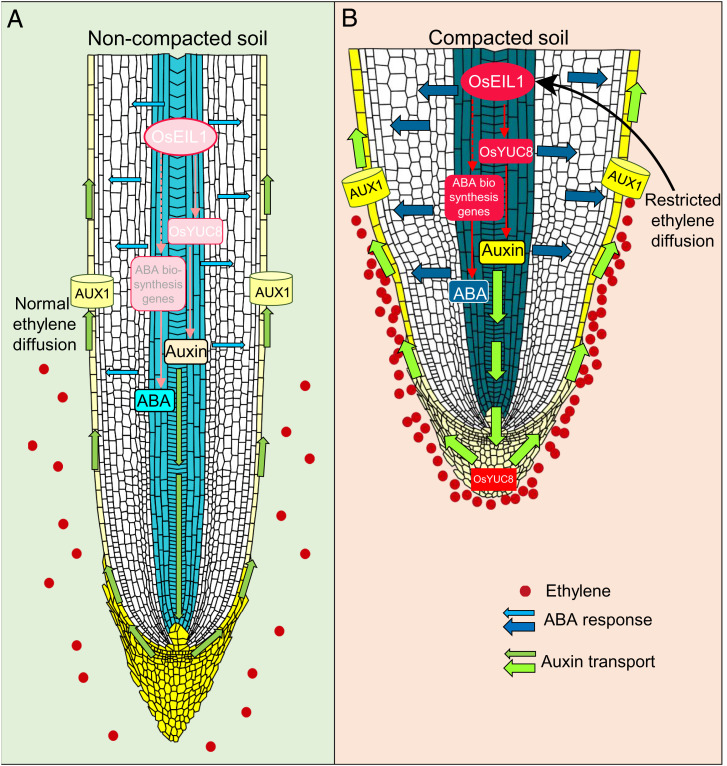
Ethylene orchestrates induction of both auxin and ABA biosynthesis to regulate inhibition of elongation and promotion of radial expansion of rice roots in compacted soil. Schematic representation of root responses in (*A*) noncompacted soil vs. (*B*) compacted soil. Compacted soil conditions induce an ethylene response due to restricted outward diffusion of ethylene through smaller air-filled soil pores. Ethylene accumulation promotes OsEIL1 activation, which directly up-regulates auxin biosynthesis through Os*YUC8*. The enhanced auxin response in epidermal cells of meristematic and elongation zones inhibits epidermal cell elongation (and thus root elongation). In parallel, ethylene signaling promotes (indirectly) higher ABA biosynthesis in vascular tissues causing radial expansion of root cortical cells, thereby also contributing to the inhibition of root elongation in compacted soil conditions. Red dots represent gaseous ethylene molecules, red ellipse indicates enhanced ethylene response in compacted soil conditions. Intensity of color (light to darker) and width of arrow indicate higher response/level for auxin (yellow) and ABA (sky blue to royal blue). Auxin transport is denoted by green arrows.

Our results provide mechanistic insights into how rice roots adapt to soil compaction via ethylene-mediated up-regulation of auxin and ABA biosynthesis and movement. Reduced ethylene diffusion in compacted soil causes this signal to accumulate in root tip tissues (10), triggering the stabilization of OsEIL1, which then up-regulates expression of *OsYUC8*-mediated auxin biosynthesis. This newly synthesized auxin is transported by the auxin carrier OsAUX1 from its site of synthesis to its target site in epidermal cells of the elongation zone. The resulting higher auxin levels trigger inhibition of epidermal cell elongation, which acts to constrain cell elongation in inner root tissues, resulting in inhibition of root elongation. However, rice roots also employ ABA as another signal to promote cortical cell radial expansion in response to soil compaction (summarized in Fig. 6).

Increased synthesis of both auxin and ABA signals in response to compaction and elevated ethylene response causes wild-type root tips to swell. Classically, root tip swelling was reasoned to facilitate root penetration of compacted soil by widening the pores in the soil (8). However, we have experimentally demonstrated that root tip swelling is attenuated in rice ethylene- and ABA-mutants, yet they exhibit improved penetration abilities in compacted soil. This suggests that roots that remain narrower are better able to penetrate compacted soil. Conversely, roots that swell in compacted soil appear less able to penetrate.

Is there any evolutionary advantage for the ABA-induced root-tip swelling response during soil compaction? It is possible that in conditions of soil compaction where the root is growing into channels/cracks but otherwise cannot penetrate, root tip swelling might play the classically presumed role of widening the channels, thereby allowing root penetration that otherwise could not occur. Future work will explore the relationship between these hormone-driven root growth responses to compaction and structural features of the soil, such as the presence of channels and cracks, thickness of the compacted soil layer and levels of moisture. Nevertheless, in our experimental system, where soil compaction is uniform, the root swelling response is not needed and in fact is negatively associated with penetration.

Soil compaction stress is also known to decrease water availability (32), which may serve to increase root ABA levels (in addition to the induction by ethylene). Although soil pores can be filled with water molecules in compacted soil conditions, the water is less accessible to roots as it is more tightly held (by greater matric forces) in smaller pores in compacted soil than in larger pores in noncompacted soil, making it more difficult for plant roots to extract (32). Indeed, soil water release curves indicate lower water potential (moisture is less accessible to the plant) of compacted than noncompacted soils at the same soil water content (33). Thus, small void size and lower water potential render the water molecules less accessible for roots in compacted soils, which is likely to mimic mild drying conditions and therefore can enhance ABA levels in root tip tissues. Hence, promotion of radial swelling by ABA may serve as an adaptive response designed to increase root surface area and facilitate water uptake during compaction stress.

## Materials and Methods

### Soil Materials.

A Newport series loamy sand (sand, 83.2%; silt, 4.7%; and clay, 12.1%; pH 6.35; organic matter 2.93%; FAO Brown Soil) was used in all soil experiments. Soil was collected from the University of Nottingham farm at Bunny, Nottinghamshire, UK (52.52°N, 1.07°W), passed through a <2 mm sieve and moisture content calculated by drying at 65 °C until constant weight.

### Plant Materials and Growth Conditions.

All rice plants for seed production were grown in paddy fields in Shanghai, China (30°N, 121°E) and Sanya, China (18°N, 109°E) in summer and winter, respectively. Seeds (Nipponbare, Hwayoung, Dongjin, *DR5:VENUS*, *osaba1*, *osaba2*, *mhz4*, *mhz5-1*, *oseil1, osein2, osein2 mhz5-1, EIN2 OE mhz5-1, osyuc8-2* (34), *DR5:VENUS/osaux1-3* and *DR5:VENUS/osein2*) were surface sterilized with 25% bleach for 10 min, then washed five times with autoclaved reverse osmosis (RO) water. The genetic backgrounds of the T-DNA insertional mutants are: *osaba1*, *osaba2, mhz4*, *mhz5-1*, *oseil1* and *osein2,* Nipponbare; *osaux1-1* and *osaux1-3*, Dongjin; *osyuc8-2*, Hwayoung. Seeds were germinated on moist sterilized filter paper for 2 d in the dark at 28 °C in a growth chamber (12-h photoperiod at 300 µmol m^−2^ s^−1^ light intensity, 70% relative humidity).

After primary root emergence (4 to 6 mm in length), seedlings were gently placed in topsoil packed at different compaction levels (1.1 g cm^−3^ and 1.6 g cm^−3^ BD for noncompacted and compacted soil conditions, respectively; the upper 1 cm contained noncompacted soil in both treatments) in a 3D printed column (33 mm diameter × 100 mm height), as previously described (10, 21). After 5 d, the primary root tips were carefully removed via washing the soil medium to keep the root tips intact for confocal imaging. Root lengths were analyzed via ImageJ (https://imagej.nih.gov/ij/).

### Expression Profiling.

Total RNA was isolated from wild-type (cv. Nipponbare) primary root tips (1 cm, encompassing the meristematic and elongation zones) of 7-d-old plants using TRIzol reagent (Applied Biosystems) as per the manufacturer’s protocol. To remove genomic DNA contamination, isolated RNA was treated with DNase1 enzyme (1 U µg^−1^ of total RNA). One microgram of total RNA from each of three independent biological replicates (each replicate comprised 10 to 15 root tips) was used for cDNA synthesis using RevertAid First-strand cDNA synthesis kit (Thermo Fisher) according to the manufacturer’s instructions.

SensiMix SYBR Hi-ROX (Bioline) master mix was used for qRT-PCR on a qTOWER real- time PCR System (Analytik Jena GmbH) with three biological replicates per treatment. Expression values were normalized with expression values of an endogenous control, ubiquitin5 (*Os01g0328400*). Relative expression levels (fold change; FC) were calculated using the 2^-Δ(ΔCt)^ method with respect to the control, as described previously (35). Primers used for rice ABA biosynthetic genes and their expression analysis are listed in *SI Appendix*, Table S1. All FCs were statistically evaluated using Student’s *t* test.

### ABA Measurement from Rice Root Tips.

For endogenous ABA quantification, wild-type (cv. Nipponbare) primary root tips (1 cm) were harvested from noncompacted and compacted soil-grown plants. The root tips were gently washed with autoclaved RO water to remove soil particles, immediately snap frozen in liquid nitrogen and stored at −80 °C. Extraction (∼8 to 10 mg fresh weight per sample) was performed according to (36); cold 10% methanol containing 1 M formic acid (vol/vol) was used as the extraction solvent. In brief, [^2^H_6_] ABA was added as an internal standard prior to extraction, and samples were then extracted using 1 mL of extraction solvent, homogenized, and purified using Oasis HLB columns (30 mg/mL, Waters Corporation). For the data in Fig. 1*A*, ABA measurements were performed by UHPLC-MS/MS using an Acquity UPLC System (Waters) equipped with an Acquity UPLC BEH C18 column (100 × 2.1 mm, 1.7 µm; Waters) coupled to a triple quadrupole Xevo TQ-S MS (Waters). For *SI Appendix*, Fig. S1*C*, analyses were conducted using a Phenomenex Polar C18 column (150 × 2.1 mm, 2.5 µm; Phenomenex), using Milli-Q water (A) and ACN (B), both with 0.02% formic acid (vol/vol) as mobile phase, with measurements performed on a LC-MS/MS system comprising a 1260 Infinity II LC System coupled with 6495 Triple Quad LC/MS System, Jet Stream and Dual Ion Funnel technologies (Agilent Technologies).

### Radial Sectioning and Confocal Imaging.

Primary roots were removed from soil columns and immediately washed with autoclaved RO water in three consecutive water-filled dishes using fine brush strokes to remove soil particles. Washing and clearing of the root tips were performed as previously described (21). One-centimeter root tips of auxin reporter lines were fixed in freshly prepared 4% PFA (Paraformaldehyde) and vacuum infiltration treatment was carried out for 2 h. Fixed root tips were washed with 1 × PBS buffer three times and kept in ClearSee solution for 24 h. Fluorescence of the cleared root tips was imaged with a TCS-SP8 confocal microscope using 10% argon and DPS1 lasers.

To obtain cross-sectional images, root tip samples (apical 1.5 cm) were embedded in 3.5% melted agarose and 100-µm-thick transverse sections were cut from the elongation zone using a Leica Vibratome, stained with Calcofluor White dye (Sigma) for 1 min on a glass slide, and imaged with a Leica SP5 confocal microscope using the UV laser.

### X-Ray CT Imaging.

Equally germinated seedlings (2 d old) of wild-type and *osaba1*, *osaba2*, *mhz4*, and *mhz5-1* mutants were grown in 3D-printed columns (33 mm diameter × 100 mm height) filled with sandy loam soil at 1.1 g cm^−3^ and 1.6 g cm^−3^ BD for noncompacted and compacted soil conditions, respectively. The plants were grown in a growth chamber at 28 °C with a 12-h photoperiod at 300 µmol m^−2^ s^−1^ light conditions and 70% relative humidity. Five-day-old seedlings were scanned using a Phoenix v|tome|x M 240 kV X-ray µCT system (Waygate Technologies [a Baker Hughes business]) at the Hounsfield Facility (University of Nottingham, Sutton Bonington Campus, UK). The scans consisted of the collection of 2,520 projection images in FAST mode (continuous rotation), with an X-ray tube energy and current of 140 kV and 200 µA, respectively. The detector exposure time was 131 msec, and the voxel resolution was 57 μm. Scan time was 5 min.

### ABA Treatments in Hydroponic and Soil Systems.

Five-day-old equally germinated seedlings (*oseil1*, *osein2*, *mhz5-1* mutants, and Nipponbare wild-type; *osaux1-1, osaux1-3* mutants, and Dongjin wild-type) were transferred to a 2 L beaker containing autoclaved RO water and 10 µM ABA (Sigma) and grown for 2 d in a growth chamber at 28 °C with a 12-h photoperiod at 300 µmol m^−2^ s^−1^ PAR and 70% relative humidity. After 48 h, primary root tips (1 cm) were harvested, washed with RO water and placed in 4% PFA for 1 h. Subsequently, root tips were washed in PBS buffer two times and thereafter cleared in ClearSee solution for 15 d. Cleared root tips were then treated with Calcofluor White dye and imaged using confocal microscopy (Leica, TCS-SP5), as described previously (21).

Sandy loam soil was packed at 1.1 g cm^−3^ BD and the soil was treated with 25 mL of 0, 10, 50, and 100 µM ABA. Germinated seedlings were placed in the soil and grown in a growth chamber at 28 °C with a 12-h photoperiod at 300 µmol m^−2^ s^−1^ PAR and 70% relative humidity. The soil columns were treated with an additional 5 mL of the respective ABA concentrations on day 2, and on day 4 primary root tips (1 cm) were harvested, washed with RO water (five times), and fixed in a 4% melted agarose block for radial sectioning, staining with Calcofluor White dye, and imaging using TCS-SP5 confocal microscopy.

### Ethylene Treatment.

Germinated seedlings (*oseil1*, *osein2*, *mhz5-1* mutants, and Nipponbare wild-type; *osaux1-1*, *osaux1-3* mutants, and Dongjin wild-type) were treated with 20 ppm ethylene gas as described previously (10). After 3 d of ethylene treatment, primary root tips (1 cm) were harvested and subsequently ten root tips from the control and ethylene treatments were cleared in ClearSee solution for 15 to 20 d for confocal imaging of longitudinal sections. The remaining five root tips per treatment were imbedded in a 3.5% agarose block for radial sectioning and imaging with SP5 confocal microscopy.

### Propidium Iodide Staining and Cell Length Analysis.

Seeds were germinated in the dark at 28 °C for 4 d, and germinated seedlings were grown in compacted and noncompacted soil for 4 d. Primary roots were harvested, cleaned with sterilized water, immersed in 1 μM propidium iodide for 3 min, and imaged using Leica Laser Scan Microscope (SP5) at an excitation wavelength of 561 nm and emission wavelengths of 600 to 650 nm. Confocal images were analyzed via ImageJ. Cell length analysis was performed as described previously (21).

### NAA and ACC Treatments.

For NAA treatment, germinated seedlings were transferred into compacted soil with or without addition of 0.1 µM NAA and grown for 5 d at 300 µmol m^−2^ s^−1^ PAR and 70% relative humidity. Seedlings were imaged using a Nikon DSLR camera. Primary roots lengths were then quantified using Image J. For ACC treatment, germinated seedlings were transferred into PCR plates without bottoms and grown in water with or without addition of 100 µM ACC for 5 d. Thereafter, seedlings were imaged using a Nikon DSLR camera, and their primary root lengths were quantified using Image J.

### In situ Hybridization.

Fresh 5-d old primary roots cultured in noncompacted and compacted soils were fixed in formalin-acetic acid-alcohol solution, dehydrated using ethanol and Clearene (Leica), and then embedded in paraffin (Sigma). The antisense and sense probes were labeled using the DIG RNA Labeling Kit (SP6/T7, Roche). The procedures for in situ RNA hybridization followed the protocol described by Dreni et al. (37). Images were obtained using a Nikon microscope (Eclipse 80i). The primer sequences were listed in *SI Appendix*, Table S2.

## Supplementary Material

Supplementary File

## Data Availability

All study data are included in the article and/or supporting information.
