# Supplementary material for: Ethylene inhibits rice root elongation in compacted soil via ABA- and auxin-mediated mechanisms
Source: Proc Natl Acad Sci U S A. 2022 Jul 18;119(30):e2201072119. doi: 10.1073/pnas.2201072119 (PMC9335218; doi:10.1073/pnas.2201072119)
Supplement: Supplementary File [file pnas.2201072119.sapp.pdf]

PNAS
www.pnas.org

Supplementary information for

Ethylene inhibits rice root elongation in compacted soil via ABA and auxin mediated mechanisms

Guoqiang Huang[1,11], Azad Kilic[2], Michal Karady[3], Jiao Zhang[1], Poonam Mehra[2], Xiaoyun Song[1], Craig J. Sturrock[2], Wanwan Zhu[1], Hua Qin[4], Sjon Hartman[5,6], Hannah M. Schneider[7], Rahul Bhosale[2], Ian C. Dodd[8], Robert E. Sharp[9], Rongfeng Huang[4], Sacha J. Mooney[2], Wanqi Liang[1], Malcolm J. Bennett[2*] Dabing Zhang[1,10*] and Bipin K. Pandey[2,11*]

[1]*Joint International Research Laboratory of Metabolic & Developmental Sciences, State Key Laboratory of Hybrid Rice, SJTU-University of Adelaide Joint Centre for Agriculture and Health, School of Life Sciences and Biotechnology, Shanghai Jiao Tong University, Shanghai, China*
[2]*Future Food beacon and School of Biosciences, University of Nottingham, LE12 5RD, UK*
[3]*Laboratory of Growth Regulators, Institute of Experimental Botany of the Czech Academy of Sciences and Faculty of Science of Palacký University, CZ-78371 Olomouc, Czech Republic*
[4]*Biotechnology Research Institute, Chinese Academy of Agricultural Sciences, Beijing 100081, China*
[5]*Plant Environmental Signalling and Development, Faculty of Biology, University of Freiburg, 79104 Freiburg, Germany.*
[6]*CIBSS – Centre for Integrative Biological Signalling Studies, University of Freiburg, 79104 Freiburg, Germany*
[7]*Centre for Crop Systems Analysis, Wageningen University & Research, The Netherlands*
[8]*Lancaster Environment Centre, Lancaster University, Lancaster, UK*
[9]*Division of Plant Science and Technology, University of Missouri, Columbia, MO, United States*
[10]*School of Agriculture, Food and Wine, University of Adelaide, Waite Campus, Urrbrae, South Australia*

[11]*Equal contribution*
Correspondence* Prof. Malcolm J. Bennett, Prof. Dabing Zhang and Dr. Bipin K. Pandey

Email: *malcolm.bennett@nottingham.ac.uk (M.J.B.), zhangdb@sjtu.edu.cn (D.Z.) or bipin.pandey@nottingham.ac.uk (B.K.P)*

This pdf file includes

Figure S1 to S18

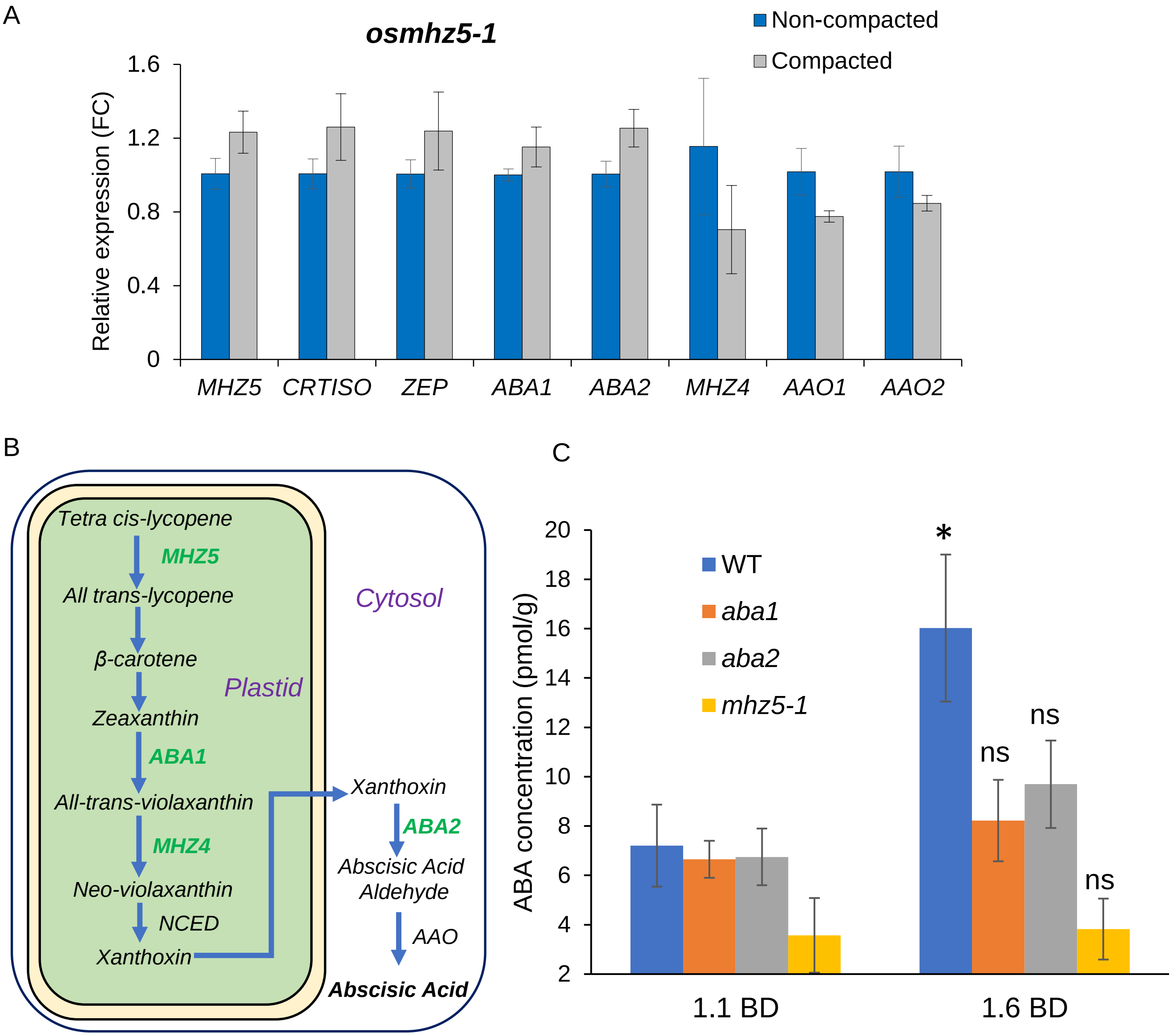

Fig. S1. Compaction is unable to induce ABA biosynthetic genes in rice *mhz5-1* mutant primary root tips. (A) Bar graph showing relative expression of ABA biosynthetic genes (*MHZ5, CRTISO, ZEP, ABA1, ABA2, AAO1 and AAO2*) in *mhz5-1* mutant root tips (1 cm) under non-compacted (1.1 g cm^{-3} BD) and compacted (1.6 g cm^{-3} BD) soil conditions. The experiment was performed for three biological replicates, each replicate comprising ~10 root tips. Data are means ± SE. (B) Schematic representation of ABA biosynthesis pathway in rice roots showing the position of enzyme-encoding genes. Loss-of-function mutants of ABA biosynthetic genes used in this study are highlighted in green font. (C) Bar graph showing ABA level (pmol g^{-1} fresh weight) in root tips of wild-type (WT; cv Nipponbare) and ABA biosynthetic mutants *aba1*, *aba2* and *mhz5-1* in non-compacted (1.1 g cm^{-3} BD) and compacted (1.6 g cm^{-3} BD) soil conditions. 3-5 replicate samples were used for ABA quantification. Each replicate (10 mg fresh weight) consisted of ~6 root tips (1 cm) in non-compacted soil and ~4 root tips in compacted soil. * represents p value ≤ 0.05, calculated by Student's *t*-test comparing genotypes between respective bulk density levels. ns, non-significant.

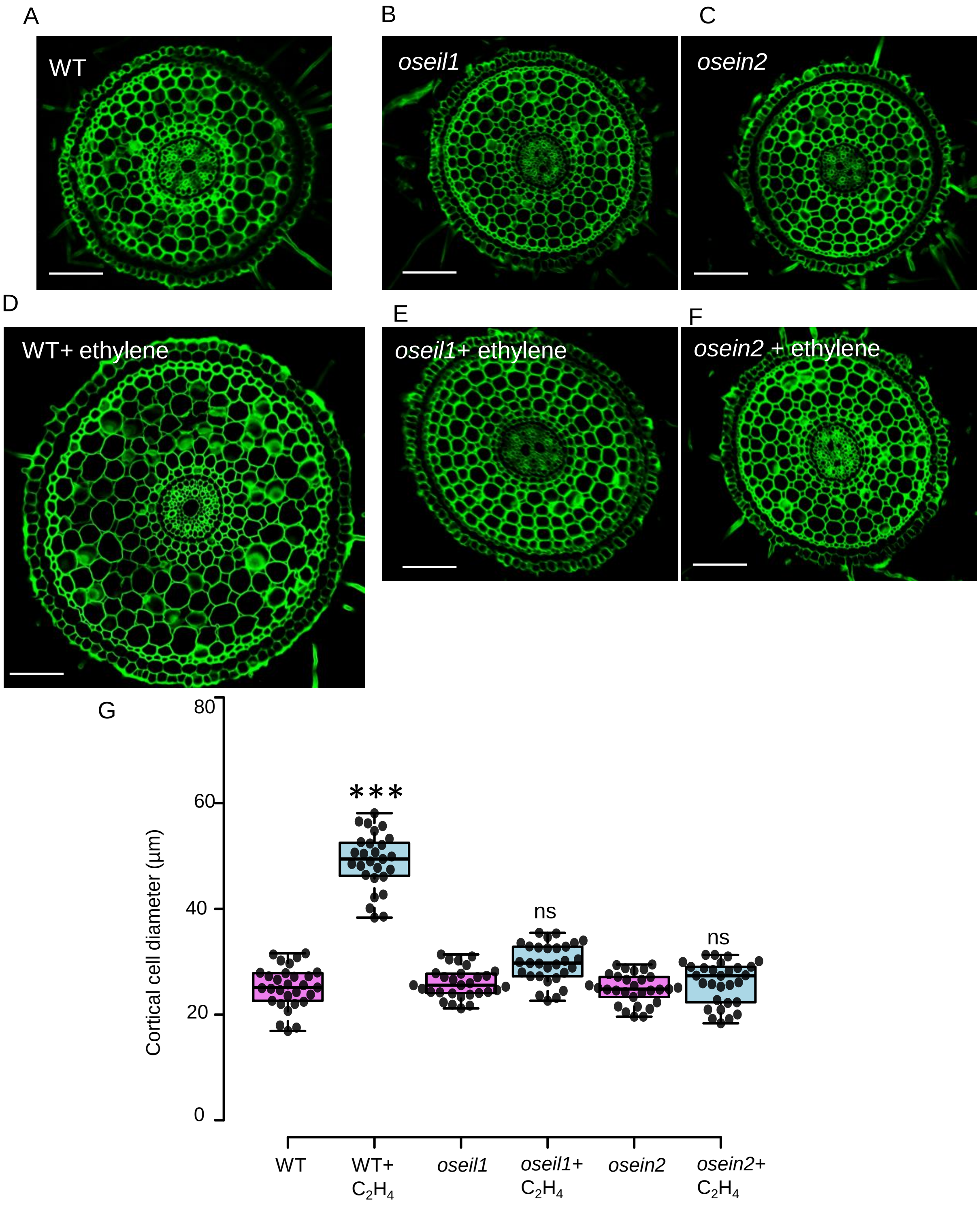

Fig. S2. Ethylene promotes the radial expansion of rice primary root cortical cells. (A-C) Representative radial section images of wild-type (WT) and, *oseil1* and *osein2* mutants as controls. (D-F) Representative images of wild-type and, *oseil1* and *osein2* after ethylene treatment (20 ppm for 3 days), showing radial expansion (diameter) of cortical cells in the wild-type but not the mutant root tips. Scale bar represents 100 µm in panels A-F. (G) Quantitative box plot showing the radial expansion (diameter) of control and ethylene-treated root tips of wild-type and, *oseil1* and *osein2* mutants. *** represents p value ≤ 0.001, calculated by Student's t-test.

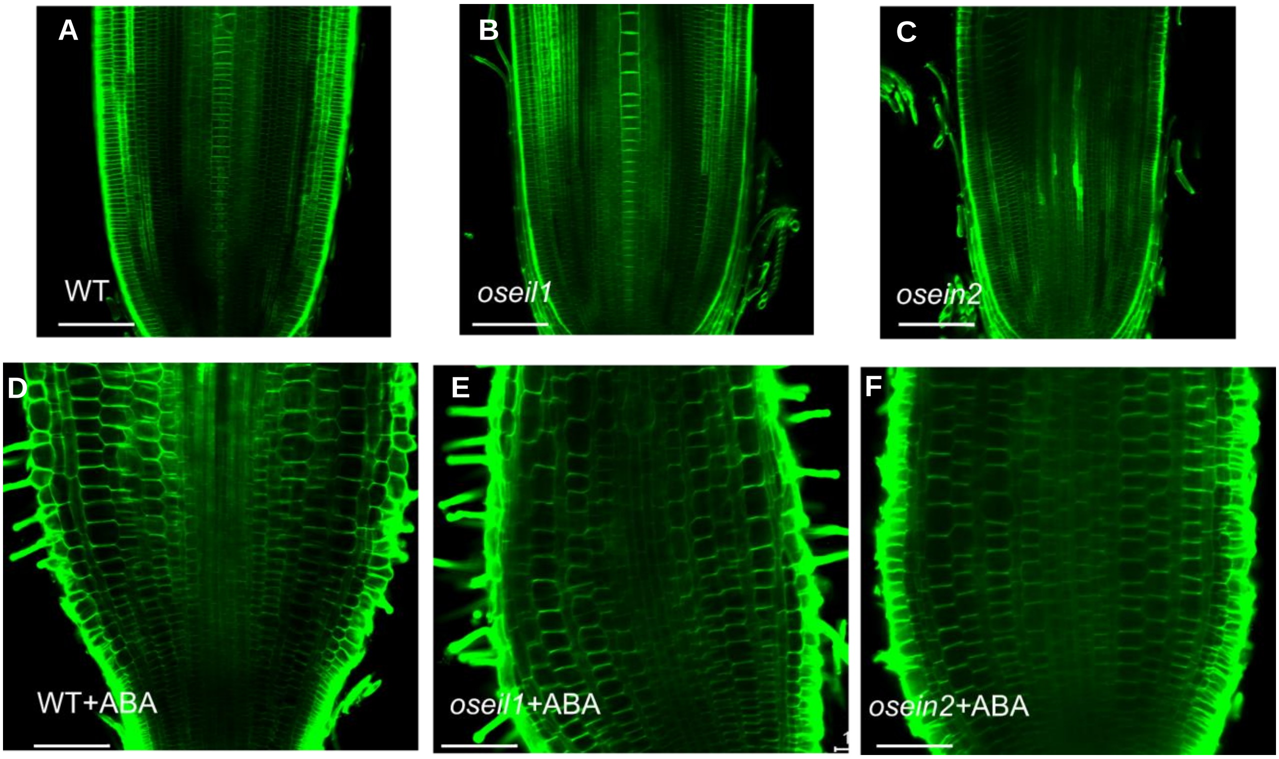

Fig. S3. ABA promotes radial expansion of rice primary root cortical cells. (A-C) Representative images of the primary root tips of wild-type (WT), *oseil1* and *osein2* mutants as controls. (D-F) Representative images of wild-type and, *oseil1* and *osein2* after ABA treatment (10 µM for 24 h), showing radial expansion of cortical cells in the root tips. Root tips were cleared using ClearSee and stained with Calcofluor stain for confocal imaging. Scale bars represent 100 µm

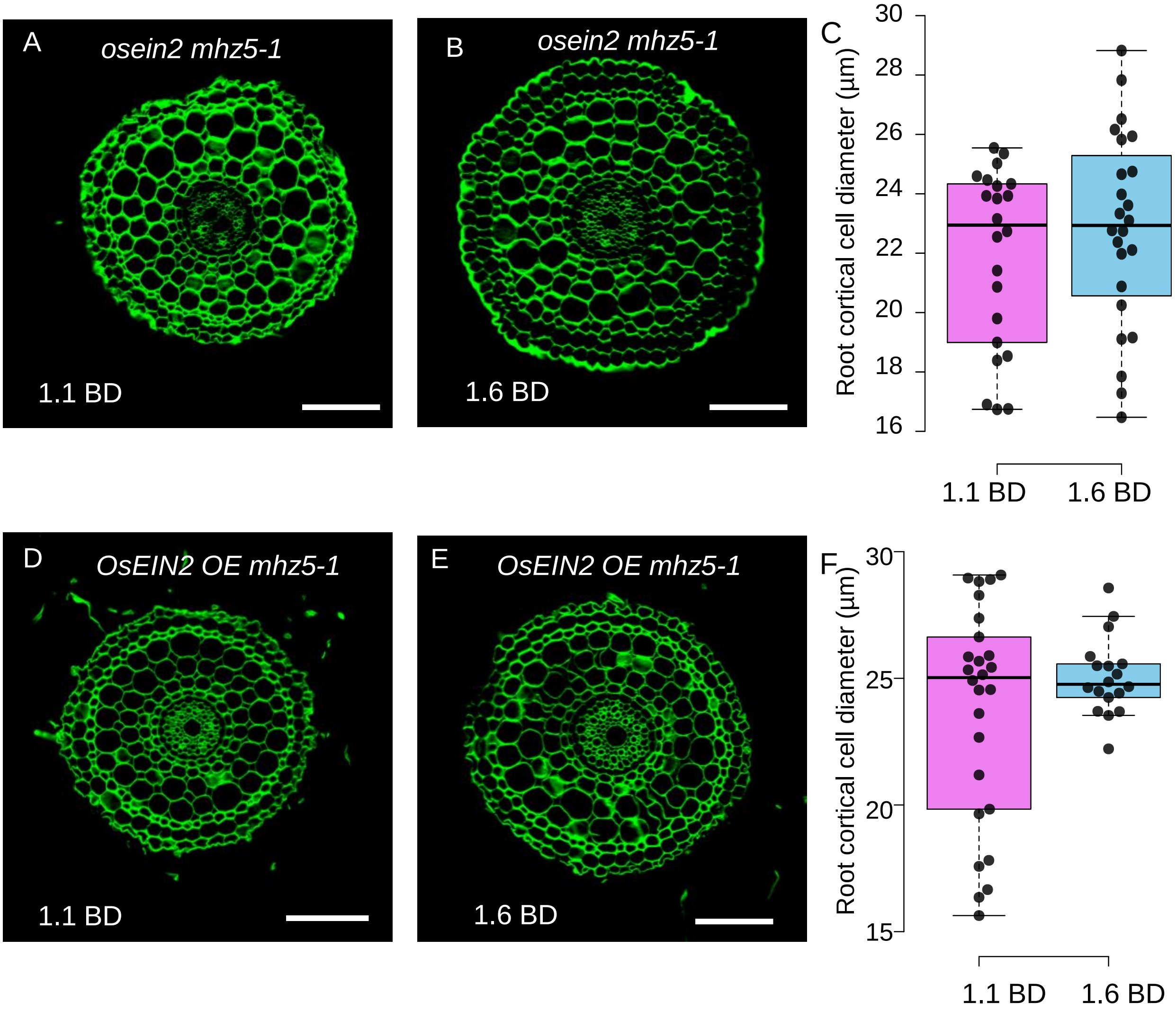

Fig. S4. ABA acts downstream to ethylene signalling to promote radial expansion of rice primary root cortical cells in compacted soil. (A-B) Representative radial section images of wild-type and *osein2 mhz5-1* double mutant in non-compacted (1.1 g cm^{-3} BD), (A) and in compacted (1.6 g cm^{-3} BD) soil (B). (C) Quantitative box plot showing the radial expansion (diameter) of *osein2 mhz5-1* root cortical cells in 1.1 g cm^{-3} BD and 1.6 g cm^{-3} BD. (D-E) Representative radial section images of the primary root tips of *OsEIN2* OE (EIN2 overexpressing line) crossed with *mhz5-1* mutant (*OsEIN2 OE mhz5-1*) in non-compacted (1.1 g cm^{-3} BD). (D) and compacted (1.6 g cm^{-3} BD) soil (E). Quantitative box plot showing the radial expansion (diameter) of *OsEIN2 OE mhz5-1* root cortical cells in 1.1 g cm^{-3} BD and 1.6 g cm^{-3} BD (F). Scale bars represents 100 µm. For each treatment >20 independent root sections were quantified from 10 primary roots, and 40-60 cortical cells were measured in each radial section. Means of all root sections were plotted in the box plots. ns > 0.05 calculated by Student's *t*-test.

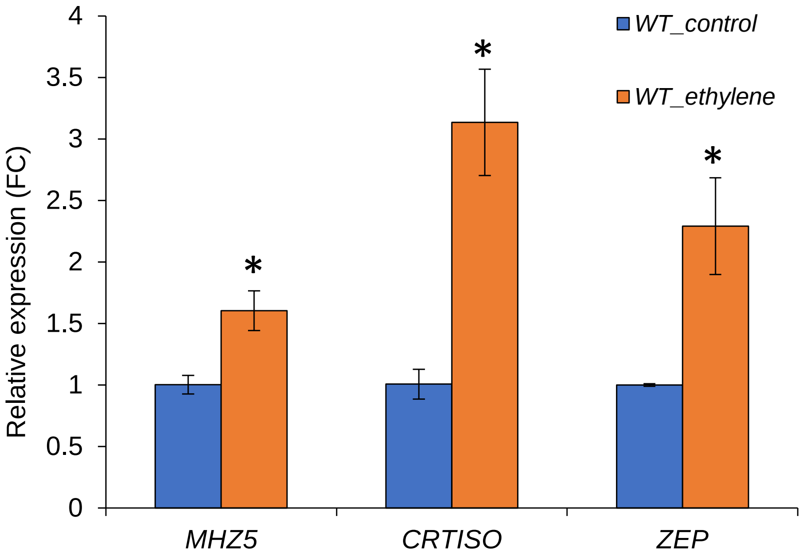

Fig. S5. Ethylene promotes induction of ABA biosynthesis genes in the rice primary root tips. Bar graph showing relative expression (fold-change, FC) of ABA biosynthetic genes (*MHZ5, CRISTO and ZEP*) in root tips (1 cm) of wild-type (WT; cv. Nipponbare) after 20 ppm ethylene treatment for 48 h compared with untreated controls. The experiment was performed for three biological replicates, each comprising ~10 root tips. Data are means ± SE. * represents *p* value ≤ 0.05, calculated by Student's *t*-test.

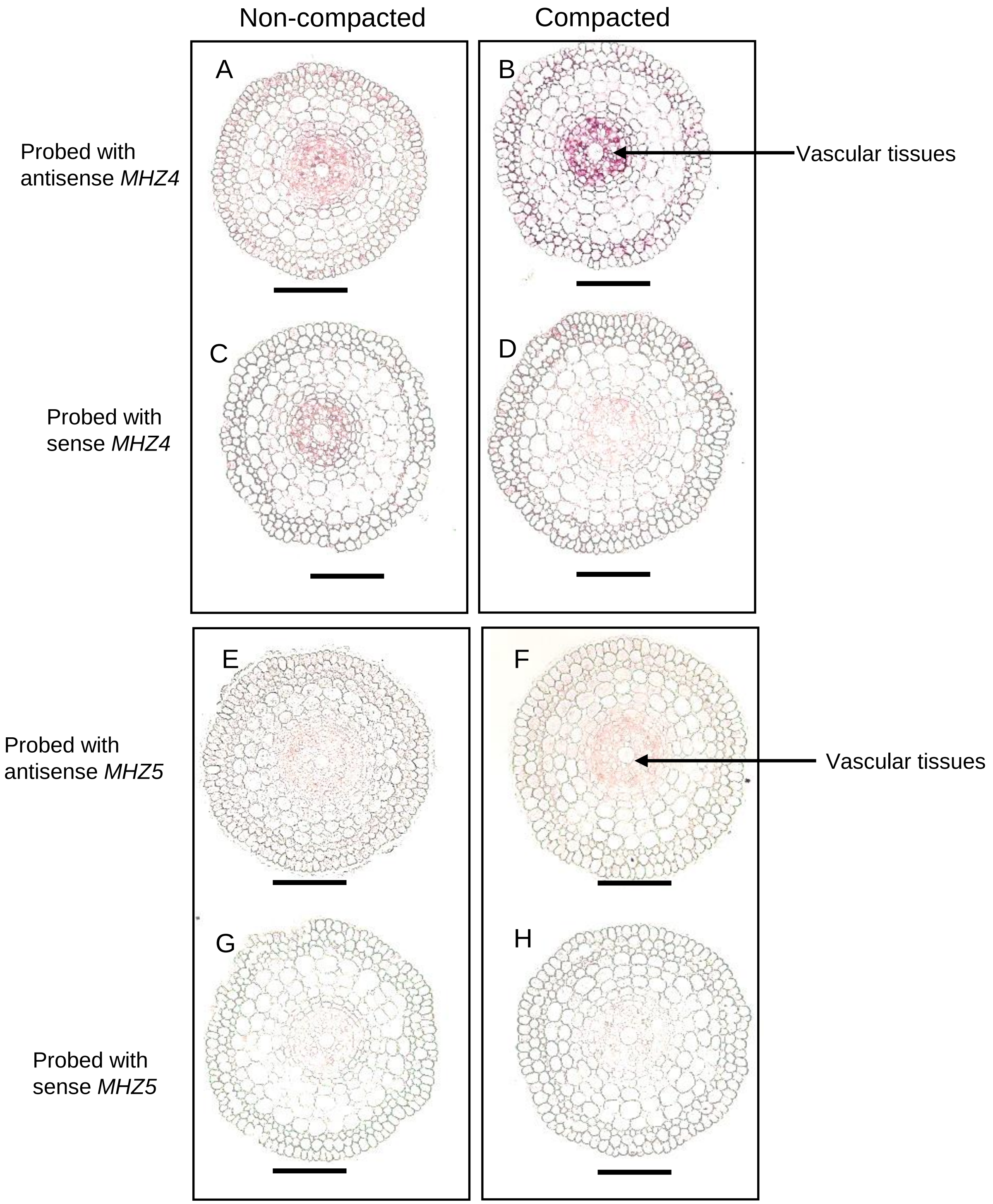

Fig. S6. Compaction induces higher expression of *MHZ4* and *MHZ5* in vascular tissues. *In Situ* Hybridisation (ISH) on radial sections of wild-type (cv Nipponbare) primary root tips of *MHZ4* anti-sense probe in non-compacted (1.1 g cm^{-3} BD) (A) and compacted (1.6 g cm^{-3} BD) (B) soil conditions. *In Situ* Hybridisation (ISH) on radial sections of wild-type (cv Nipponbare) primary root tips of *MHZ4* sense probe in non-compacted (C) and compacted (D) soil conditions. Lower panel showing ISH on radial sections of wild-type (cv Nipponbare) primary root tips of *MHZ5* anti-sense probe in non-compacted (1.1 g cm^{-3} BD) (E) and compacted (1.6 g cm^{-3} BD) (F) soil conditions. *In-Situ* Hybridisation (ISH) on radial sections of wild-type (cv Nipponbare) primary root tips of *MHZ5* sense probe in non-compacted (G) and compacted (H) soil conditions. Scale bars represent 50 µm.

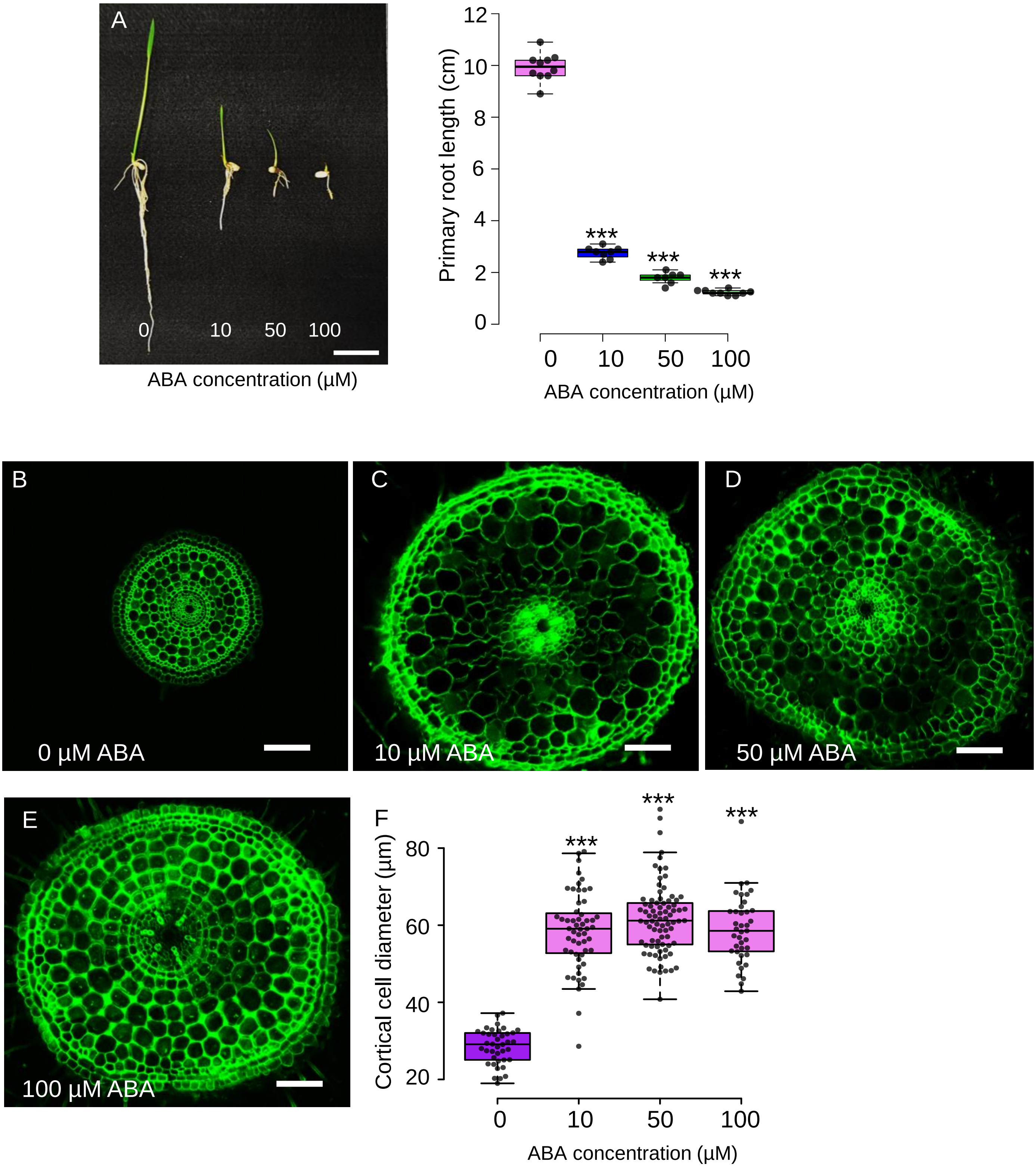

Fig. S7. ABA promotes cortical cell expansion in rice primary root tips. (A) Representative images of control (0 μM ABA) and ABA treated (10 μM, 50 μM and 100 μM ABA for 48 h) wild-type (cv Nipponbare) rice seedlings grown in non-compacted (1.1 g cm^{-3} BD) soil and their primary root lengths plotted as box plots. Scale bar represents 15 mm. (B-E) Representative radial section images of primary root tips (elongation zone) of control (0 μM ABA) and ABA treated (10 μM, 50 μM and 100 μM ABA for 48 h) wild-type (cv Nipponbare) seedlings grown in 1.1 g cm^{-3} BD soil showing radial expansion of root cortical cells. Scale bars represent 100 μm. (F) Quantitative box plot showing the radial expansion (diameter of cortical cells) of ABA-treated compared with untreated root tips. *** represents *p* value ≤ 0.0001 calculated by Student's *t*-test

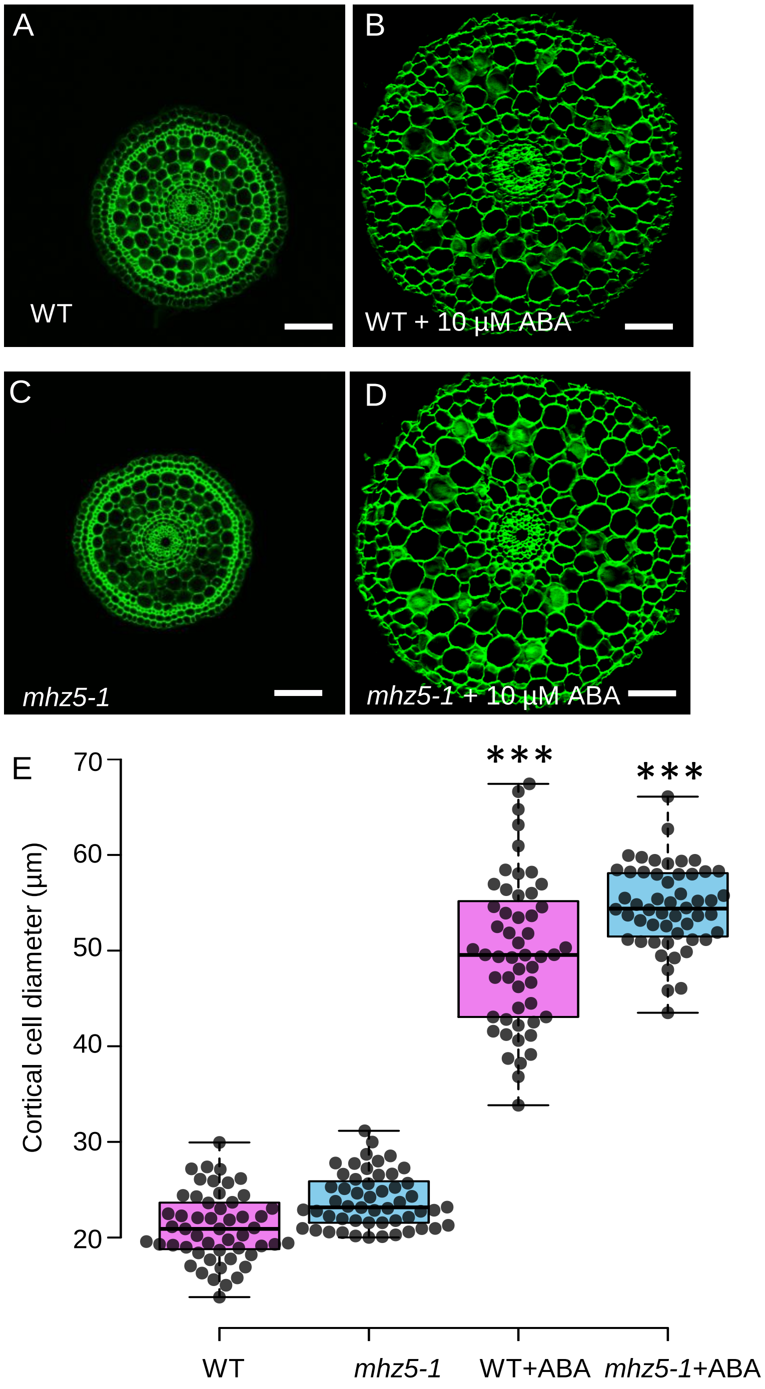

Fig. S8. *mhz5-1* is sensitive to external ABA treatment in soil. Representative radial section images of the primary root tip (elongation zone) in control (0 µM ABA) (A) and ABA treated (10 µM for 4 days) seedlings of wild-type (Nipponbare) and *mhz5-1* mutant (C, D) grown in non-compacted (1.1 g cm^{-3} BD soil). Scale bars represent 100 µm. (E) Box plot showing the radial expansion of cortical cells of WT and *mhz5-1* mutant treated with 10 µM ABA for 4 days. *** represents p value ≤ 0.0001, calculated by Student's t-test between genotypes in compacted compared with non-compacted conditions.

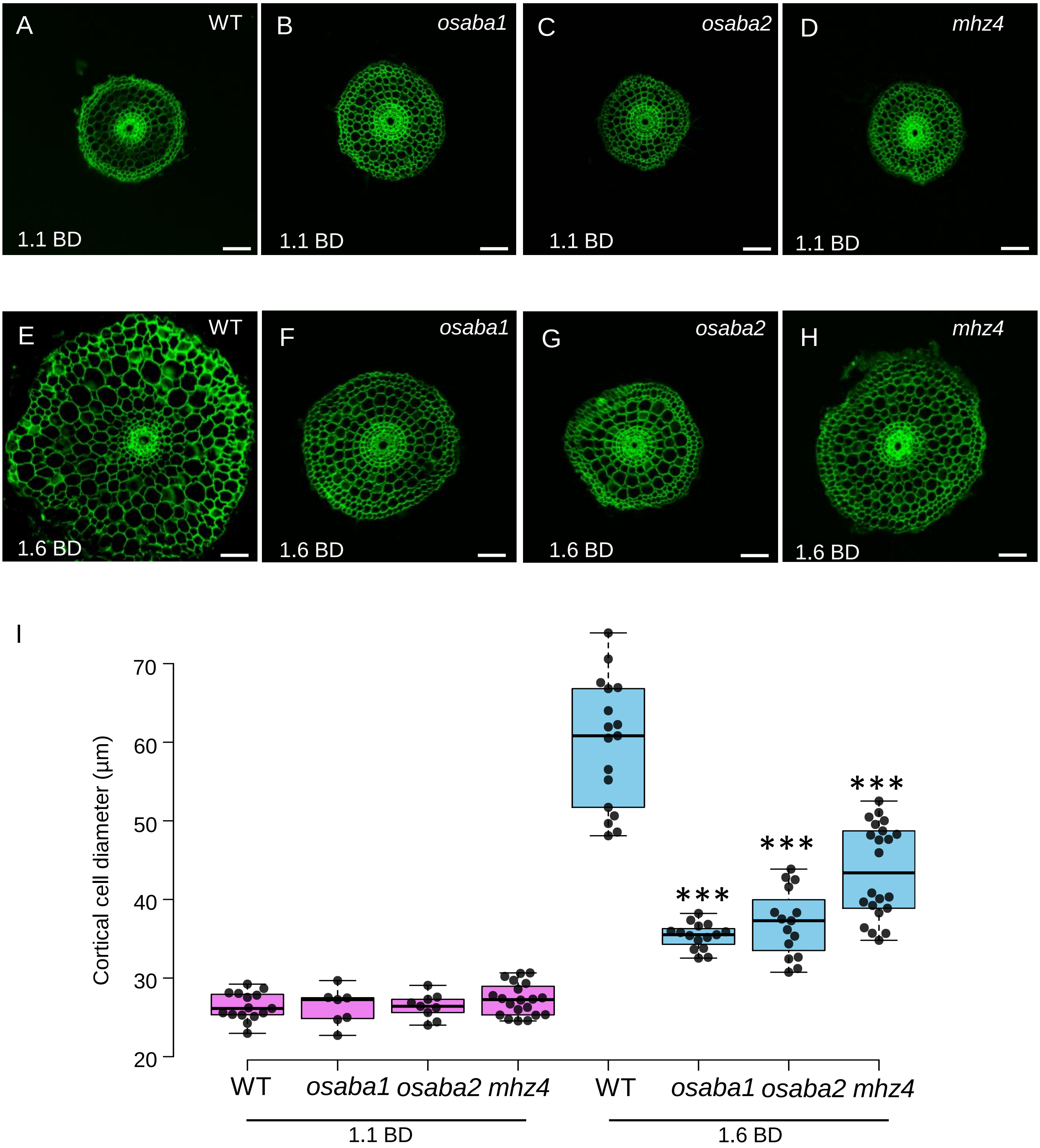

Fig. S9. Rice ABA biosynthesis mutants exhibit less radial expansion of primary root tip cortical cells in compacted soil. Representative radial section images of primary root tips of wild-type (WT) and *osaba1*, *osaba2* and *mhz4* mutants in (A-D) non-compacted (1.1 g cm^{-3} BD) and (E-H) compacted (1.6 g cm^{-3} BD) soil. Scale bars represent 100 µm. (I) Quantitative box plot showing the radial expansion (diameter) of root cortical cells in primary root tips of wild-type and *osaba1*, *osaba2* and *mhz4* mutants in non-compacted and compacted soil. *** represents p value ≤ 0.0001, calculated by Student's t-test between genotypes in compacted compared with non-compacted conditions.

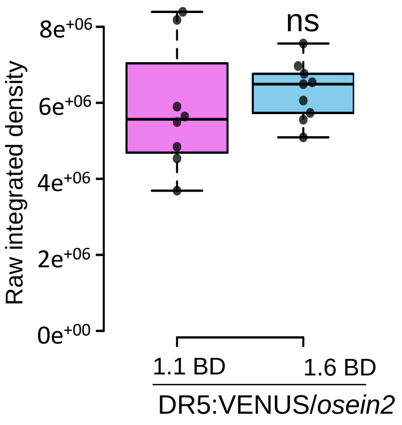

Fig. S10. Soil compaction-induced higher auxin response is abolished in *osein2* mutant roots. Box plot showing the quantitative VENUS signal in the primary root tip (including cap, meristematic and elongation zones) of DR5:VENUS/*osein2* seedlings grown in non-compacted (1.1 g cm^{-3} BD) and compacted (1.6 g cm^{-3} BD) soil. For each treatment, 8-10 root tips were used to quantify the VENUS signals. The experiment was repeated three times. ns represents p value> 0.05 calculated by Student's t-test.

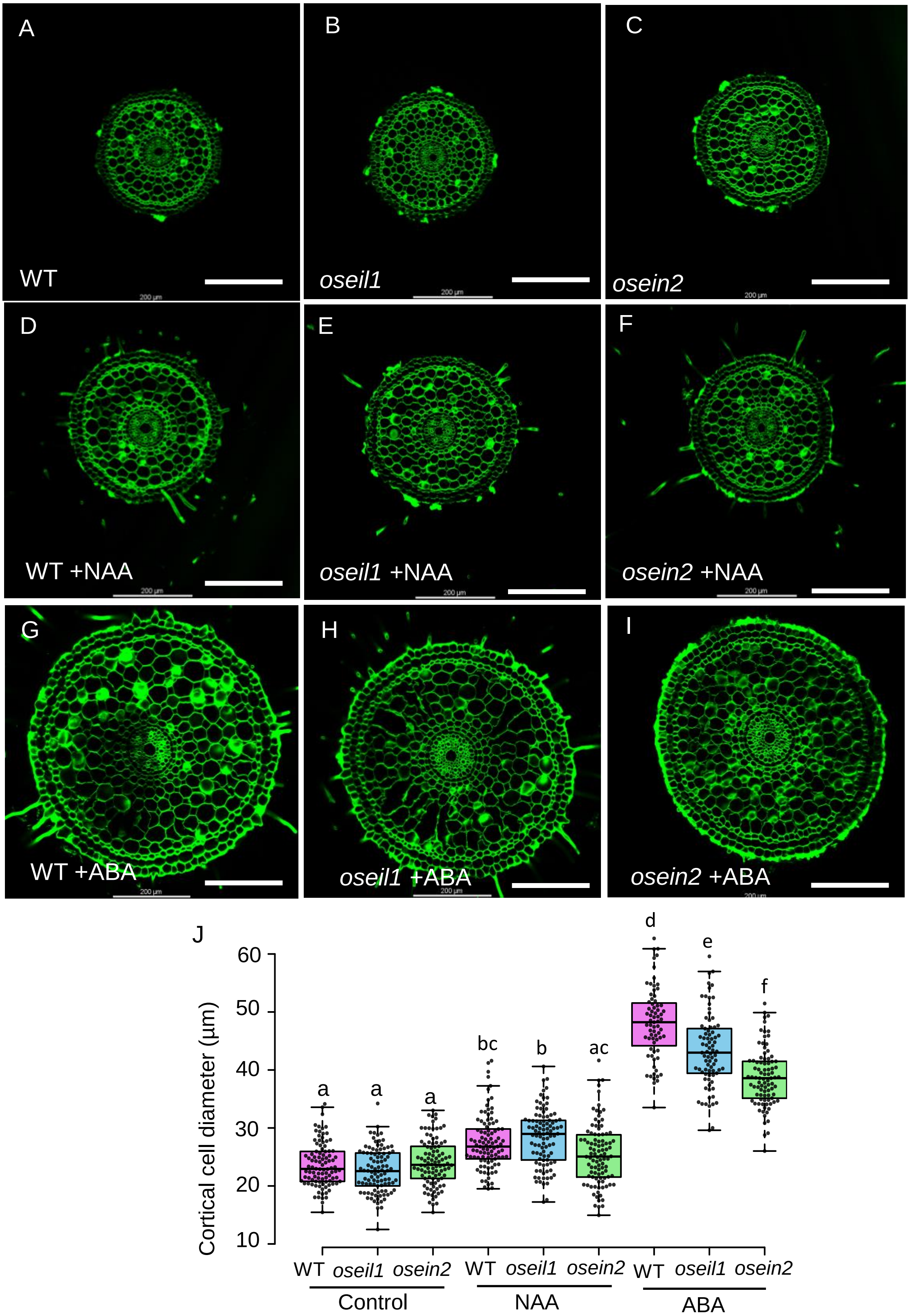

Fig. S11. Auxin does not act as primary driver for radial swelling (A-C) Representative radial section images of wild-type (WT; cv Nipponbare) (A) *oseil1* (B) and *osein2* root tips (C). (D-F) Representative radial section images of wild-type (WT; cv Nipponbare) (D) *oseil1* (E) and *osein2* (F) root tips treated with 0.01 µM NAA for 48 hours. (G-I) Representative radial section images of wild-type (WT; cv Nipponbare) (G) *oseil1* (H) and *osein2* (I) root tips treated with 10 µM ABA for 48 hours. Scale bars in panels A-I represent 200 µm. (J) Quantitative box plot showing cortical cell diameters with and without 0.01 µM NAA treatment in the root tips of wild-type, *oseil1* and *osein2* mutant root tips. Different letters represent significant differences ($p \leq 0.01$) among different genotypes and treatments using Tukey's HSD test.

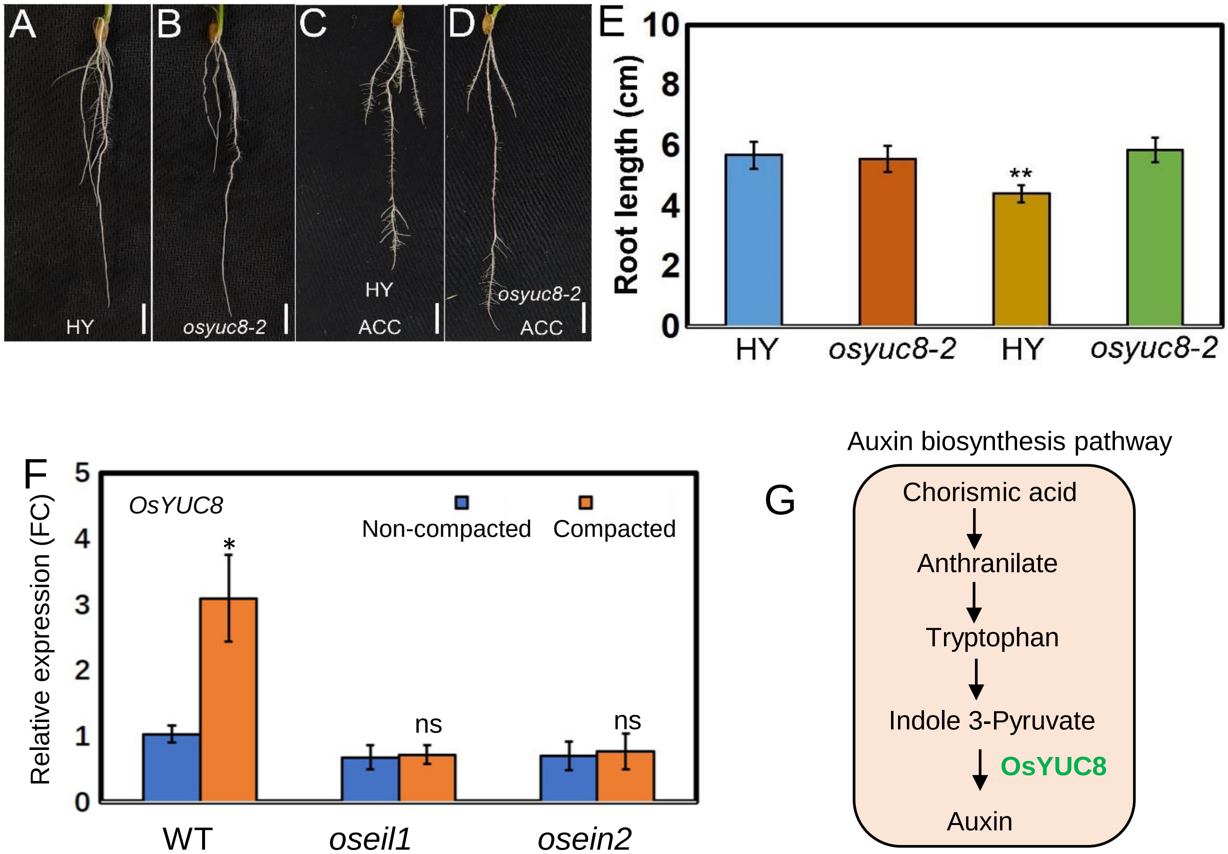

Fig. S12. The *osyuc8* mutant is not responsive to treatment with ACC (ethylene precursor). (A–D) Phenotypes of wild-type (cv. Hwayoung [HY]) (A, C) and *osyuc8-2* (B, D) primary roots grown in water without (A, B) and with (C, D) addition of 100 μM ACC. Scale bars represent 1 cm. (E) Lengths of HY and *osyuc8-2* primary roots grown with or without 100 μM ACC treatment. Data are means ± SD, n = 10. ** represents significant differences between genotypes across the treatments ($p < 0.01$, Student's *t*-test). (F) Relative *OsYUC8* expression in wild-type (WT;(cv Nipponbare) and *oseil1* and *osein2* mutant roots grown in non-compacted (1.1 g cm^{-3} BD) and compacted (1.6 g cm^{-3} BD) soils. Data are means ± SE from three independent replicates. * indicates significant differences ($p \leq 0.01$, Student's *t*-test). ns, non-significant. (G) Schematic pathway of auxin biosynthesis in rice roots showing the position of OsYUC8 where it catalyses the oxidative decarboxylation of Indole 3-Pyruvate (IPA) to produce IAA (auxin).

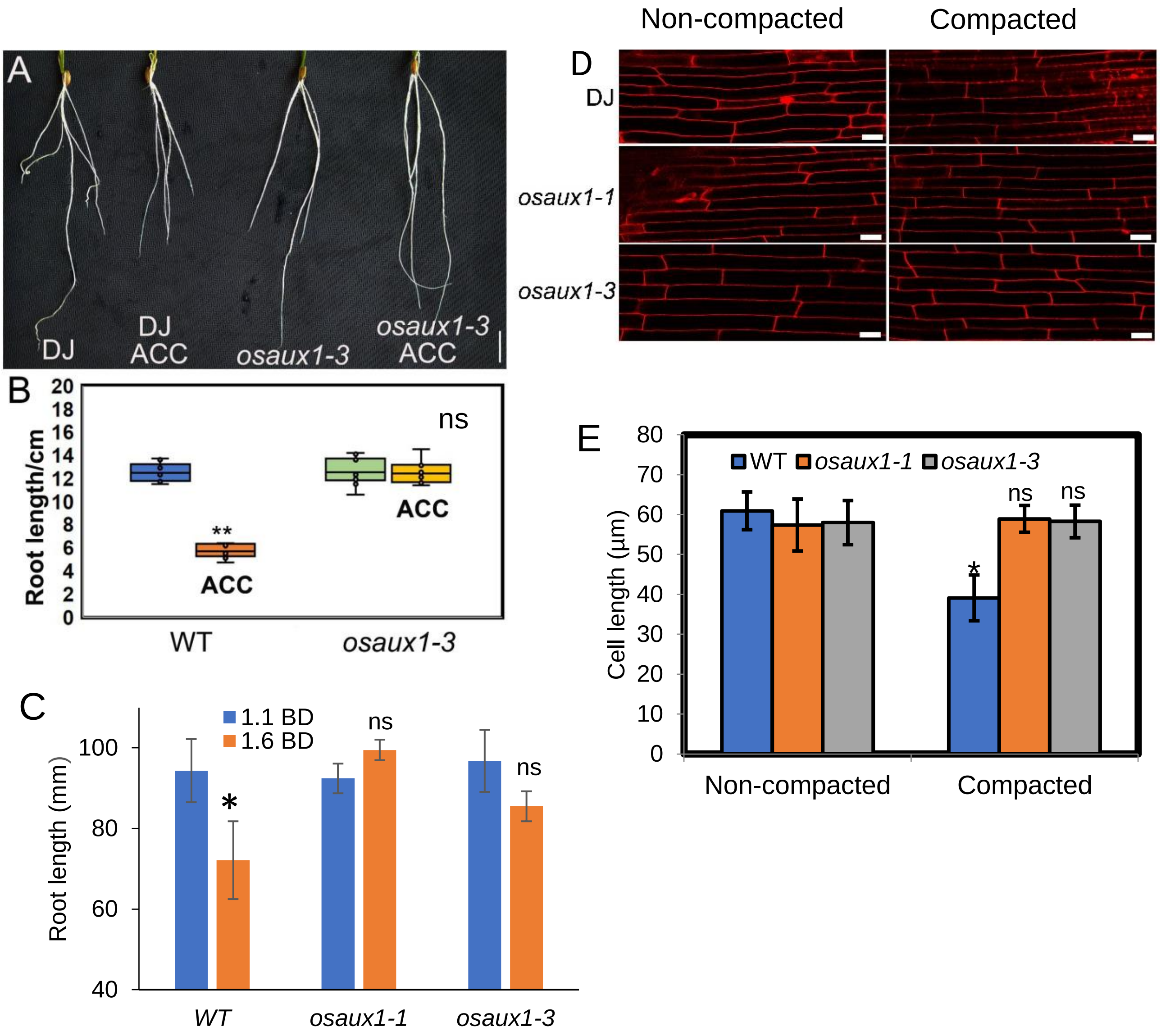

Fig. S13. The *osaux1-3* mutant is resistant to external application of ACC and soil compaction. (A) Phenotypes of wild-type (WT; cv Dongjin [DJ]) and *osaux1-3* mutant roots with or without treatment with 100 μM ACC in water. (B) Primary root length of wild-type and *osaux1-3* roots with or without treatment with 100 μM ACC. Data are means ± SD, n = 10. ** indicates significant differences between treatments ($p \leq 0.01$, Student's *t*-test). (C) Primary root lengths of wild-type and *osaux1-1* and *osaux1-3* roots grown in non-compacted (1.1 g cm^{-3} BD) and compacted soils (1.6 g cm^{-3} BD) calculated from four independent CT images. Data are means ± SD. * represents *p* value ≤ 0.05 determined by Student's *t*-test. ns, non-significant. (D) Epidermal cells in the elongation zone of DJ (Wild-type), *osaux1-1*, and *osaux1-3* roots grown in noncompacted and compacted soils. Bars, 20 μm. The epidermal cell walls were stained with 1 μM propidium iodide. (E) Epidermal cell lengths of DJ, *osaux1-1*, and *osaux1-3* roots grown in non-compacted and compacted soils. Results are given as mean ± SD, n = 10. * indicates significant differences ($p \leq 0.01$, Student's *t*-test).

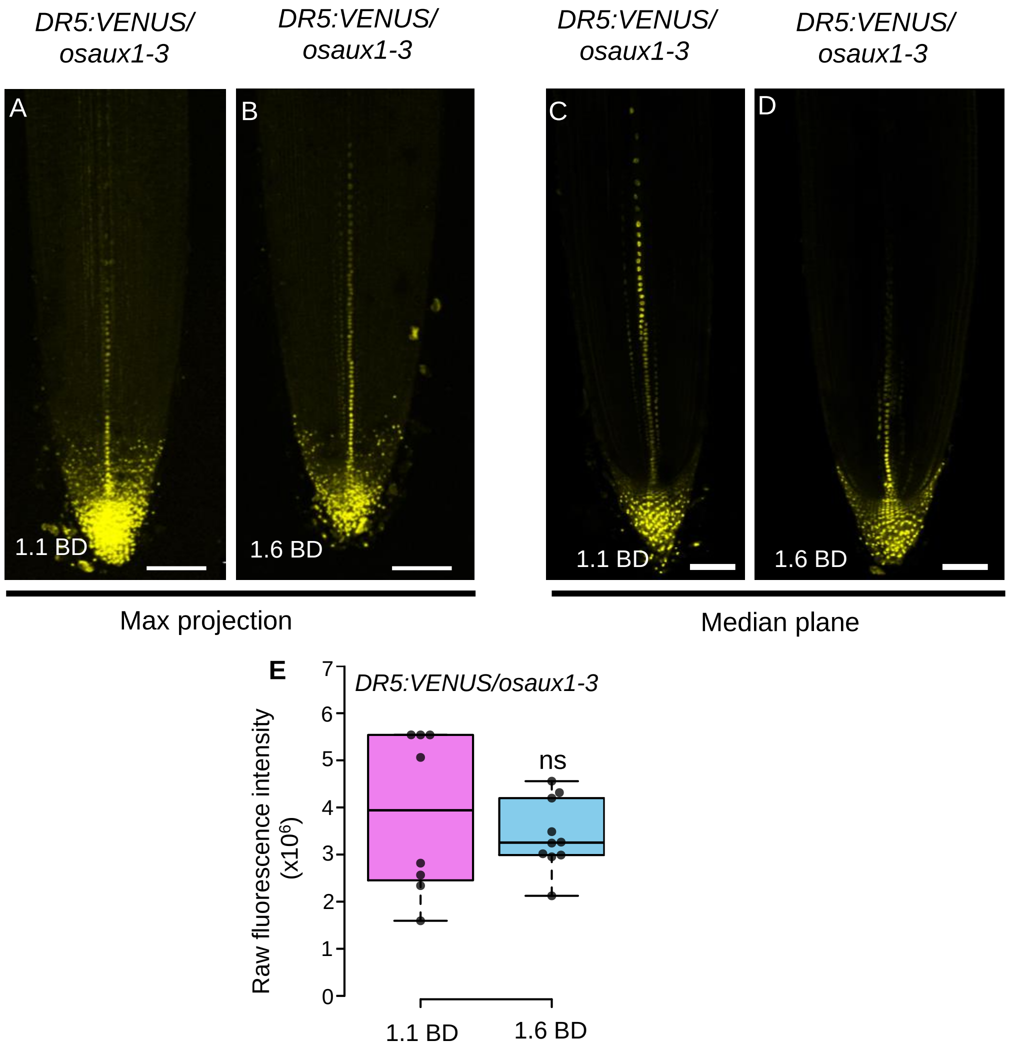

Fig. S14. *OsAUX1* is required to mobilize auxin from root tip to elongation zone to regulate inhibition of root elongation in compacted soil. (A-B) Representative confocal images (maximum projection) of *DR5:VENUS/osaux1-3* primary roots grown in (A) non-compacted (1.1 g cm^{-3} BD) and (B) compacted (1.6 g cm^{-3} BD) soil. (C-D) Representative confocal images (median plane view) of *DR5:VENUS/osaux1-3* primary roots grown in (C) non-compacted (1.1 g cm^{-3} BD) and (D) compacted (1.6 g cm^{-3} BD) soil. Scale bars represent 100 µm. (E) Quantitative box plot showing expression of *DR5:VENUS* in non-compacted and compacted soil in the *osaux1-3* mutant background. For each treatment, 8-10 root tips were analysed to calculate fluorescence intensity. The experiment was repeated three times. ns, *p* value > 0.05. calculated by Student's *t*-test.

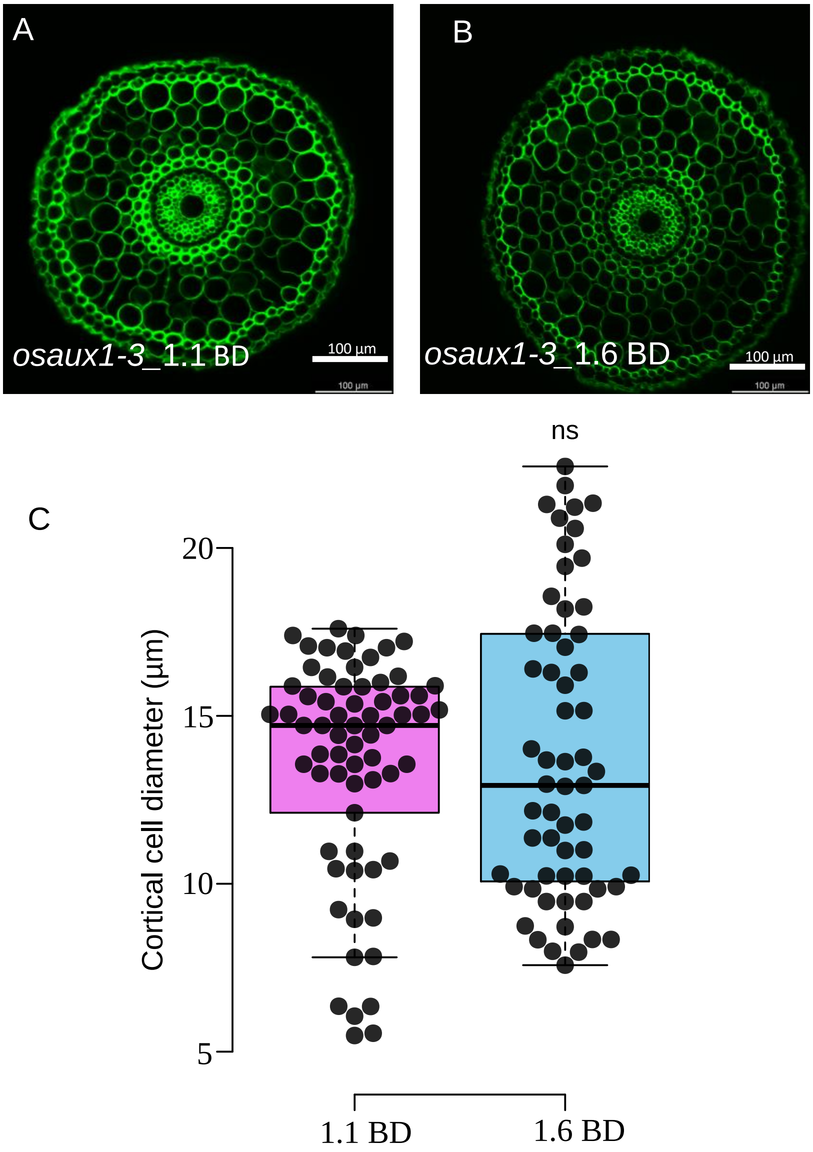

Fig. S15. *osaux1-3* mutant does not show cortical cell radial expansion under compacted soil conditions. (A, B) Representative radial section images showing unaltered cortical cell radial expansion in the elongation zone of primary roots growing in non-compacted (1.1 g cm^{-3} BD) compared with compacted (1.6 g cm^{-3} BD) soil. Scale bars represent 100 µm. (C) Quantitative box plot showing cortical cell diameters of *osaux1-3* roots in 1.1 and 1.6 g cm^{-3} BD. ns, $p > 0.05$, calculated by Student's *t*-test. n= 40-50 cells from at least four independent primary roots.

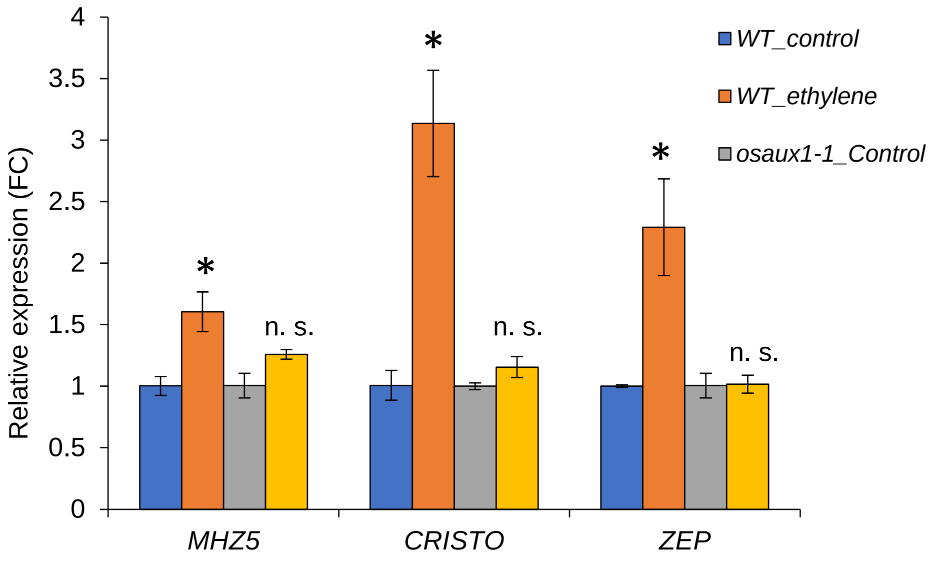

Fig. S16. Ethylene is unable to promote induction of ABA biosynthesis genes in the *osaux1-1* mutant primary root. Bar graph showing relative expression (fold-change, FC) of ABA biosynthetic genes (*MHZ5*, *CRTISO* and *ZEP*) in root tips (1 cm) of wild-type (WT; cv. Dongjin) and ethylene-insensitive *osaux1* mutant after treatment with 20 ppm ethylene for 48 h. Relative expression was calculated with respect to untreated wild-type roots. The experiment was performed in three biological replicates, each comprising ~10 root tips per genotype and treatment. * represents p value ≤ 0.05, calculated by Student's t-test.

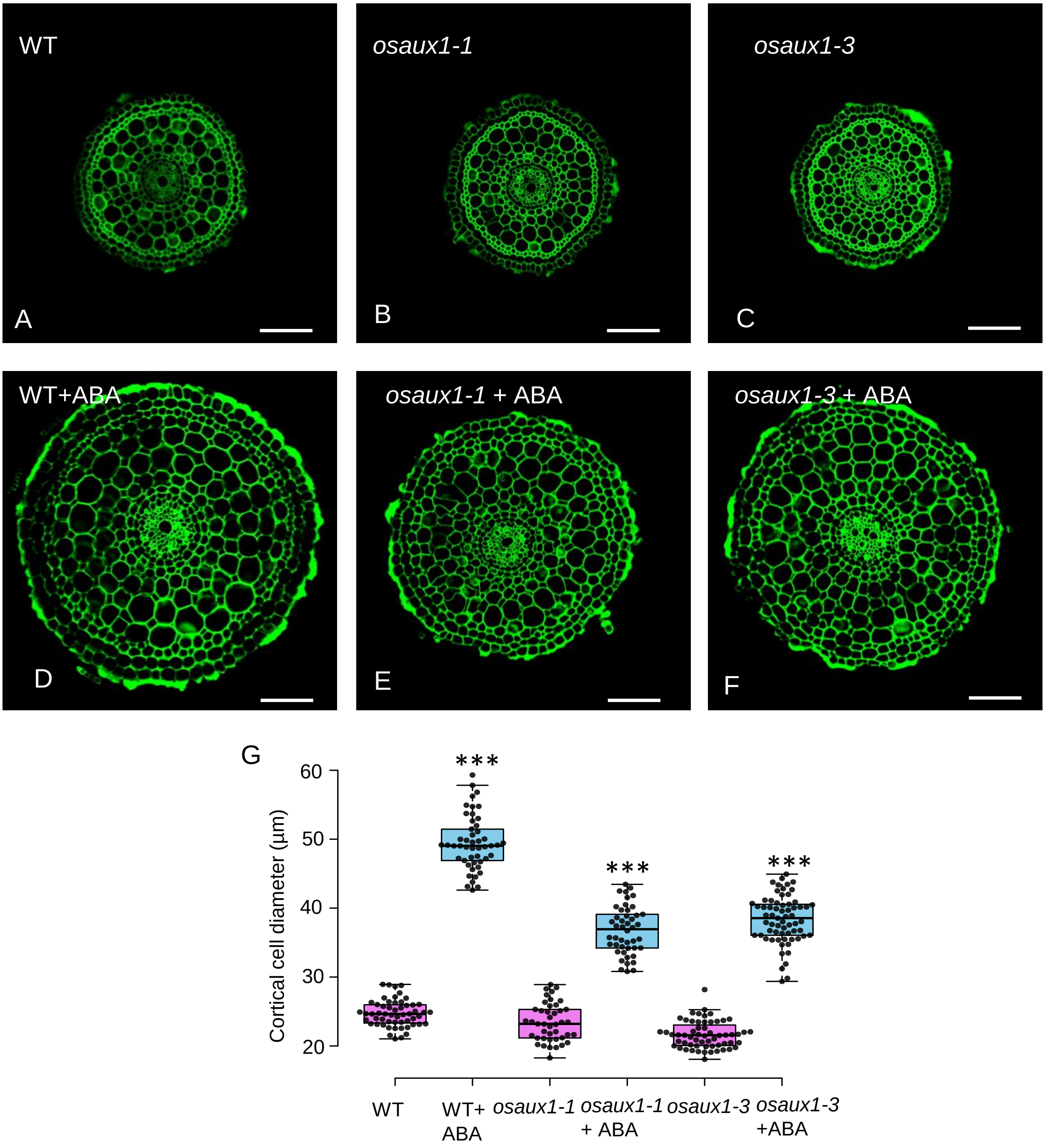

Fig. S17. ABA acts as a downstream signal to auxin to promote the radial expansion of *osaux1* (auxin resistant1) mutants primary root cortical cells. (A) Representative radial section images of wild-type (WT, cv Dongjin) and *osaux1-1* and *osaux1-3* mutants as controls. (B) Representative images of wild-type and *osaux1-1* and *osaux1-3* mutants after ABA treatment (10 µM for 24 h) showing radial expansion of cortical cells in the root tips. Scale bars in panels A-B represent 100 µm. (G) Quantitative box plot showing cortical cell diameters with and without ABA treatment in the root tips of wild-type and *osaux1-1* and *osaux1-3* mutants. *** represents p value of ≤ 0.001, calculated by Student's t-test.

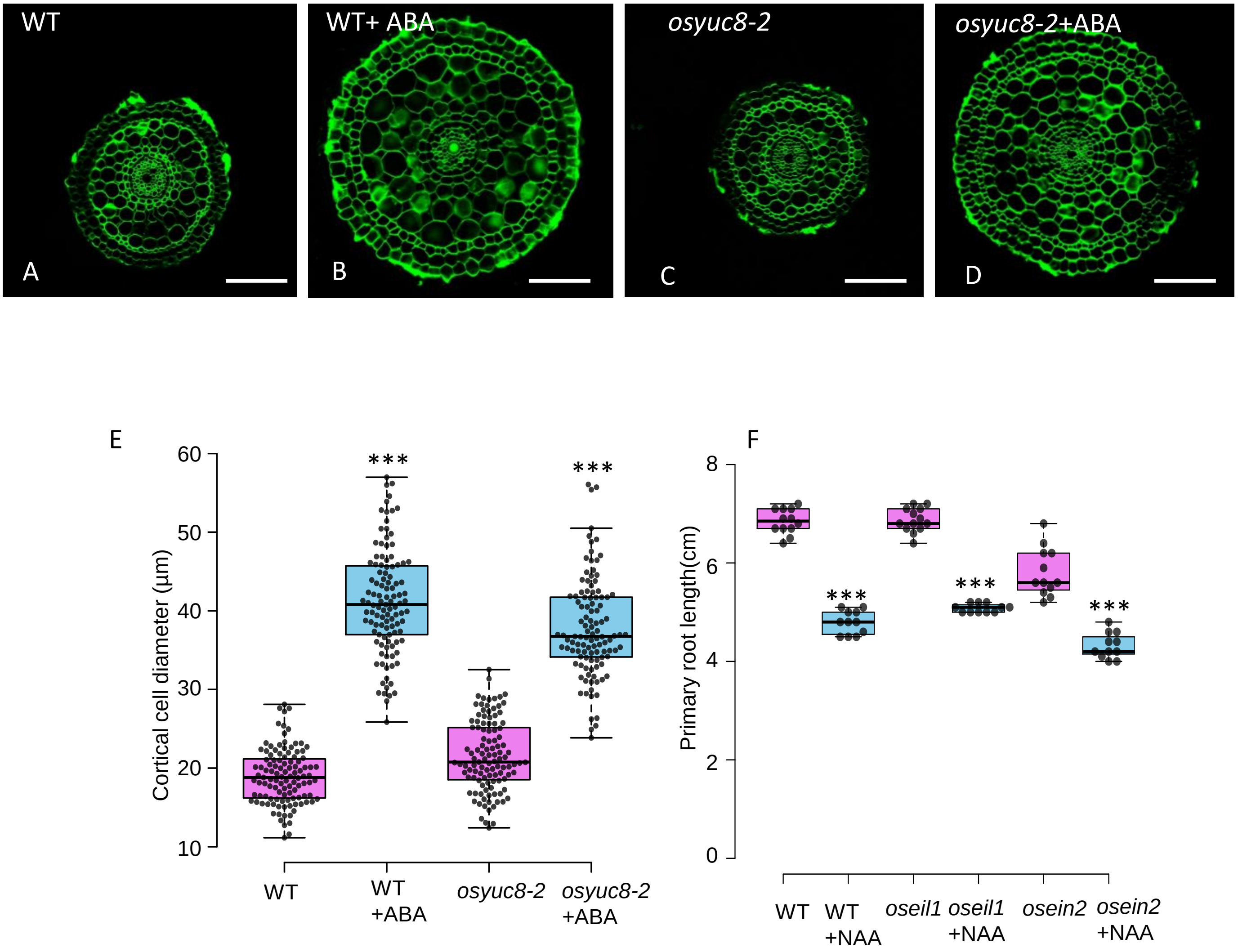

Fig. S18. ABA acts as a downstream signal to auxin and promote the radial expansion of *osyuc8-2* (auxin biosynthetic mutant) primary root cortical cells. (A-B) Representative radial section images of wild-type (WT; cv Hwayoung) untreated (A) and treated with 10 µM ABA (B) for 48 hours. (C-D) Representative radial section images of *osyuc8-2* untreated (C) and treated with 10 µM ABA (D) for 48 hours. Scale bars in panels A-D represent 100 µm. (E) Quantitative box plot showing cortical cell diameters with and without ABA treatment in the root tips of wild-type and *osyuc8-2* mutant. (F) Quantitative box plot showing primary root length with and without 0.01 µM NAA treatment in the root tips of wild-type, *oseil1* and *osein2* mutant root tips. *** represents *p* value of ≤ 0.0001, calculated by Student's *t*-test.
